# Establishment of a Common Marmoset Lineage Carrying a Frameshift Mutation in *SETD1A*, a Schizophrenia Risk Gene

**DOI:** 10.1111/jnc.70546

**Published:** 2026-09-03

**Authors:** Zefeng Wei, Harumi Nakao, Yuichi Hiraoka, Maria Harbers, Yukiko Abe, Motoki Goto, Moe Tamano, Mai Saeki, Yusuke Kishi, Atsu Aiba

**Affiliations:** ^1^ Laboratory of Animal Resources, Center for Disease Biology and Integrative Medicine, Graduate School of Medicine The University of Tokyo Tokyo Japan; ^2^ Laboratory of Molecular Neurobiology, Institute for Quantitative Biosciences The University of Tokyo Tokyo Japan; ^3^ Laboratory of Molecular Biology, Graduate School of Pharmaceutical Sciences The University of Tokyo Tokyo Japan; ^4^ Division of Aging Biology, Research Institute for Science and Technology Tokyo University of Science Chiba Japan

## Abstract

Appropriate histone modifications are essential for maintaining functional chromatin structure and gene expression, and dysfunction of their regulators has been linked to a variety of diseases. Among these modifications, trimethylation of lysine 4 on histone H3 (H3K4me3) is a well‐characterized epigenetic mark enriched at transcription start sites of actively transcribed genes. H3K4me3 regulates gene transcription by recruiting transcription factors, facilitating chromatin accessibility, and preventing DNA methylation. In mammals, methylation of H3K4 is catalyzed by a family of histone methyltransferases including SET domain containing 1A (SETD1A), which is primarily responsible for genome‐wide deposition of H3K4me2/3. Loss‐of‐function variants in *SETD1A*, highlighting its critical role in brain development and cognitive function, are strongly associated with schizophrenia (SCZ) and other neurodevelopmental disorders, but the underlying mechanisms remain largely unclear. To better understand the epigenetic and neurobiological consequences of *SETD1A* dysfunction, non‐human primate models can serve as a useful tool because of their close evolutionary relationship to humans and highly developed cognitive abilities. In this study, we established a genetically engineered common marmoset ( *Callithrix jacchus* ) lineage carrying a frameshift mutation in *SETD1A*, which is, to the best of our knowledge, the first non‐human primate lineage carrying a mutation in an epigenetic regulatory gene associated with SCZ, and confirmed germline transmission of the mutant allele. In a comparison between fibroblasts derived from one *SETD1A* mutant and one wild‐type marmoset, the mutant showed a lower SETD1A protein level, modest differences in H3K4me3 deposition, and broader differences in gene expression profiles. Although these molecular observations require validation using additional biological replicates, the establishment of this *SETD1A* mutant marmoset lineage provides a valuable platform for bridging molecular mechanisms with primate neurobiology and for investigating the role of epigenetic regulation in the pathophysiology of neuropsychiatric and neurodevelopmental disorders.

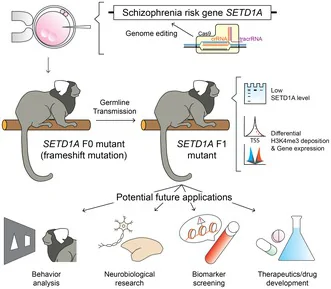

## Graphical abstract

The study describes the establishment of a genetically engineered common marmoset ( *Callithrix jacchus* ) lineage harboring a frameshift mutation in *SETD1A*, an epigenetic regulatory gene associated with schizophrenia, and confirms germline transmission of the mutant allele. Comparison of fibroblasts from one *SETD1A* F1 mutant and one wild‐type marmoset revealed lower SETD1A protein levels, modest differences in H3K4me3 deposition, and broader differences in gene expression profiles. Overall, this *SETD1A* mutant marmoset lineage provides a valuable platform for investigating the *SETD1A*‐associated diseases.

**Abbreviations**
DDG: differentially H3K4me3‐deposited gene
DEG: differentially expressed gene
H3K4me3: trimethylation of lysine 4 on histone H3
KMT2: histone‐lysine‐N‐methyltransferase 2
RRID: Research Resource Identifier
SCZ: schizophrenia
TSS: transcription start site

## 1 Introduction

Genomic DNA is organized into chromatin in eukaryotic cells. Chromatin structure directly determines the accessibility of genomic regions and thereby regulates gene expression. The fundamental repeating unit of chromatin is the nucleosome, which consists of an octamer of four core histones (H2A, H2B, H3, and H4) wrapped by approximately 147 base pairs of DNA. Post‐translational modifications of core histones can alter chromatin structure and enable distinct cell types to express different sets of genes from the same genome (reviewed in Millán‐Zambrano et al. 2022). The trimethylation of lysine 4 on histone H3 (H3K4me3) is one of the most extensively studied histone modifications that is commonly found at transcription start sites (TSSs). H3K4me3 activates gene transcription by recruiting basal transcription factor (Lauberth et al. 2013; Vermeulen et al. 2007), facilitating chromatin accessibility (Flanagan et al. 2005; Sims et al. 2005; Wysocka et al. 2006), and preventing DNA methylation (Ooi et al. 2007). In mammals, mono‐, di‐, and trimethylation of H3K4 are catalyzed by the histone‐lysine‐N‐methyltransferase 2 (KMT2) family. All members of the KMT2 family function as the catalytic subunit of an evolutionarily conserved histone methyltransferase complex known as the Complex of Proteins Associated with Set1 (COMPASS; Shilatifard 2012).

It is well known that dysregulated histone modification can result in a wide range of diseases, and mutations in each KMT2 family member (*Mll1*, *Mll2*, *Mll3*, *Mll4*, *SETD1A*, and *SETD1B*) are associated with distinct clinical phenotypes (Vallianatos and Iwase 2015). Haploinsufficiency of SET domain containing 1A (*SETD1A*), a KMT2 family member primarily responsible for the genome‐wide H3K4me2/3 deposition, is strongly associated with schizophrenia (SCZ; Singh et al. 2016; Takata et al. 2014, 2016) as well as with other neurodevelopmental disorders characterized by intellectual disability, impaired language development, early‐onset epilepsy, and autism spectrum disorder (Kummeling et al. 2021; Yu et al. 2019).

Studies using heterozygous *Setd1a*‐deficient mice have revealed the importance of *Setd1a* in working memory and sociability (Mukai et al. 2019; Nagahama et al. 2020); nevertheless, the utility of rodent models is limited by the relative simplicity of rodent behavioral repertoires and the substantial evolutionary distance between rodents and primates. To overcome these limitations, a novel non‐human primate model carrying *SETD1A* mutations analogous to those in human patients would be a powerful tool for assessing higher order cognitive functions and complicated social behaviors, as well as for identifying translational biomarkers with greater relevance to humans. In this study, we utilized the common marmoset ( *Callithrix jacchus* ), a small, prolific, and tractable New World monkey. We established a novel marmoset lineage carrying a *SETD1A* mutation and examined SETD1A protein expression and H3K4me3 deposition in the mutant. To the best of our knowledge, this is the first genetically engineered non‐human primate lineage carrying a mutation in an epigenetic regulatory gene associated with SCZ. Because loss‐of‐function variants in human *SETD1A* markedly increase the SCZ risk with an odds ratio of 35.2 (Singh et al. 2016) and are under strong negative selection (according to the Genome Aggregation Database v4.1.0), this genetically engineered marmoset lineage is expected to serve as a valuable and highly reproducible platform for investigating epigenetic regulation in primates as well as pathophysiology of the associated diseases, although further validation in additional animals will be required as the *SETD1A* mutant colony expands.

## 2 Materials and Methods

### 2.1 Ethics Statement

All experiments in this study were reviewed and permitted by the Institutional Animal Care and Use Committee of the University of Tokyo (permission number M‐P20‐083 and A2025M006) and conducted following “Manual for Animal Experiment of the University of Tokyo” as well as “Guidelines for the Care and Use of Non‐human Primates in Neuroscience Research” of The Japan Neuroscience Society. The study was also carried out in compliance with the ARRIVE (Animal Research: Reporting of In Vivo Experiments) guidelines for animals.

### 2.2 Animals

Common marmosets were purchased from CLEA Japan Inc. or bred at the University of Tokyo. Marmosets were maintained under a 12‐h light/dark cycle with food and water available ad libitum, and the ambient temperature was maintained at 28°C. Marmosets were housed in cages measuring 390 × 560 × 700 mm (standard cages) and were able to communicate visually and vocally with animals housed in neighboring cages. Newborn marmosets were housed with their mothers and siblings until 3 months of age. From 3 to 12 months of age, marmosets were group‐housed in standard cages (3–4 animals per cage) or a large cage (7–10 animals per cage; width, 1840–2635 mm; depth, 1800–3000 mm; height, 2100–2200 mm) with age‐matched individuals from different litters. After 12 months of age, marmosets were housed individually in standard cages. All cages were provided with environmental enrichment, including wooden sticks.

### 2.3 Design and Validation of CRISPR/Cas9 Target Sites

Potential CRISPR/Cas9 target sites in the 5′ region of exon 6 from marmoset *SETD1A* were analyzed using CRISPRdirect (RRID: SCR_018186), and the site with the fewest predicted off‐target sites (5′‐CACAGCCATCGCCGCCACCA‐3′) was used. For the in vitro digestion assay (IDA), DNA fragments amplified from marmoset *SETD1A* by polymerase chain reaction (PCR; 5′‐GCCCTGGGACCCAGGATGCTC‐3′ and 5′‐CAGGAGGCCGGTAGTCCTGGTC‐3′) using KOD FX *Neo* (TOYOBO, cat. no. KFX‐201) were digested by SpCas9 (Guide‐it Recombinant Cas9; Clontech, cat. no. 632641)/crRNA (Integrated DNA Technologies, IDT)/tracrRNA (IDT) complex. For validation using marmoset embryonic stem (ES) cells (CMES40, RRID: CVCL_B946; RIKEN BRC CELL BANK, cat. no. AES0166), SpCas9/crRNA/tracrRNA complex was transfected into the marmoset ES cells using CRISPRMAX transfection reagent (Thermo Fisher Scientific, cat. no. CMAX00015), and the genomic DNA was extracted 4 days later. The target locus was amplified using the same primers as in IDA. The PCR products were cloned into plasmid vectors using TOPO TA Cloning Kit (Thermo Fisher Scientific, cat. no. 450641) and analyzed by direct sequencing (Eurofins).

### 2.4 Generation of Mutant Marmosets

The generation of mutant marmosets by autologous embryo transfer was performed as previously described (Abe et al. 2021). We prepared a SpCas9 protein/crRNA/tracrRNA mixture in Opti‐MEM medium (Thermo Fisher Scientific, cat. no. 11058021) containing 0.1% polyvinyl alcohol (PVA; Millipore, cat. no. P8136) for the injection of marmoset embryos. The mixture of SpCas9 protein/crRNA/tracrRNA was injected into the collected embryos in M2 medium (Millipore, cat. no. M7167). Details of embryo manipulation for each individual are provided in Table S2. Injected embryos were transferred autologously into the oviducts of the animals that provided the embryos. One month after embryo transfer, pregnancy was confirmed by ultrasonography.

### 2.5 Genotyping of Mutant Marmosets

Marmosets were genotyped by amplicon sequencing as previously described (Harbers et al. 2025). Genomic DNA was extracted from their hair follicles. The first PCR for *SETD1A* locus was performed under the same conditions used for the validation of the gRNA, using Illumina adaptor attached primers. The second PCR and the sequencing were conducted by SeibutsuGiken Inc. The obtained sequencing data was subjected to polymorphism analysis using CRISPResso2 (ver. 2.0.34; Clement et al. 2019). The frequency of sequences was recalculated after excluding variants with frequencies below 3%, which were considered sequencing errors.

### 2.6 Isolation of Primary Fibroblasts From Marmosets

A fragment of approximately 7–8 mm of neonatal marmoset tails was cut into 1–2 mm pieces with scissors and treated with 300 U/mL collagenases (Wako, cat. no. 03822361) at 37°C for 90 min. The collagenase‐treated tissue was ground with the gasket part of the syringe, and the tissue solution was collected by straining the cells through a 40‐μm cell strainer. The cells were then suspended in Dulbecco's Modified Eagle Medium (DMEM; Millipore, cat. no. 04429765) supplemented with 10% Fetal bovine serum (FBS; Cell Culture Technologies, cat. no. SH30071.03) and 1× penicillin/streptomycin (Nacalai Tesque, cat. no. 09367–34), seeded onto 60‐mm dishes, and cultured at 37°C in 5% CO_2_.

### 2.7 Western Blot Analysis

All replicate lanes in Western blot analysis were prepared using whole protein or histone protein extracted from the same culture dishes. Tris (cat. no. 35406‐91), HCl (cat. no. 18321‐05), NaCl (cat. no. 31320‐05), EDTA (cat. no. 15111‐45), Triton X‐100 (cat. no. 35501‐15), MgCl_2_ (cat. no. 20909‐55), Tween‐20 (cat. no. 28353‐85) were purchased from Nacalai Tesque. For analysis of SETD1A protein levels, fibroblasts were lysed in buffer containing 20 mM Tris–HCl (pH 7.4), 150 mM NaCl, 2 mM EDTA, 1% Triton X‐100 and protease inhibitors (cOmplete Mini, EDTA‐free; Roche, cat. no. 11836170001) followed by sonication. Lysates were centrifuged at 13 000*g* for 5 min, and the supernatants were quantified using a BCA assay kit (Thermo Fisher Scientific, cat. no. 23227). 25 μg of protein was separated by SDS‐PAGE on 4%–20% polyacrylamide gels (Bio‐Rad, cat. no. 4561095) and transferred onto Immobilon‐P membranes (Millipore, cat. no. IPVH00010) at 30 V overnight. For analysis of histone methylation levels, fibroblasts were lysed in buffer containing 20 mM Tris–HCl (pH 7.4), 150 mM NaCl, 0.5% Triton X‐100, and protease inhibitors (cOmplete Mini, EDTA‐free). Cells were then centrifuged at 700*g* for 10 min, and the resulting pellets were treated with 0.2 M HCl overnight. Extracted histone proteins were centrifuged at 13 000*g* for 5 min, and the supernatant was quantified using the BCA assay kit. 2 μg of histone protein was separated by SDS‐PAGE on 15% polyacrylamide gels and then transferred to Immobilon‐P membranes at 30 V for 1 h. After each antibody incubation, membranes were washed three times for 10 min each with washing buffer containing 50 mM Tris–HCl (pH 7.4), 200 mM NaCl, 1 mM MgCl_2_, 0.05% (w/v) Tween‐20. Before each primary antibody incubation, membranes were incubated for 30 min at room temperature with blocking buffer consisting of washing buffer supplemented with 5% (w/v) skim milk (BD Difco, cat. no. 232100).

The following primary antibodies were used in this study: anti‐SETD1A (Cell Signaling Technology, cat. no. 50805; 1:1000), anti‐LMNB1 (MBL Life Science, cat. no. PM064; 1:7000), anti‐H3K4me3 (Millipore, RRID: AB_1977252; 1:1000), and anti‐histone H3 (Abcam, RRID: AB_302613; 1:10 000). Membranes were probed with respective primary antibodies followed by anti‐rabbit HRP‐conjugated secondary antibodies. Bound antibodies were visualized with ECL Prime detection reagents (GE Healthcare, cat. no. RPN2232). The blots were imaged using ChemiDoc imaging systems (Bio‐Rad). The data were quantified using Image Lab software (Bio‐Rad).

### 2.8 Off‐Target Analysis

Genomic DNA from fibroblasts derived from #630 was used for whole genome sequencing, which was conducted by Rhelixa Inc. Sequencing libraries were prepared using TruSeq PCR free DNA Library Prep (Illumina) and sequenced by Illumina NovaSeq X Plus. For each sample, 600 million paired‐end reads of 150 bp were generated. Low‐quality bases or adapter sequences were eliminated by Trimmomatic (RRID: SCR_011848; version 0.38) with following options: ILLUMINACLIP:(path to adapter.fa):2:30:10 LEADING:20 TRAILING:20 SLIDINGWINDOW:4:15 MINLEN:36. The trimmed reads were aligned to the reference genome *Callithrix jacchus* ‐3.2 (caljac3) using Bowtie2 (RRID: SCR_016368; version 2.4.4). After removing duplicated reads using Picard (RRID: SCR_006525; version 2.20.4), single nucleotide variants (SNVs) and short indels were called using Freebayes (RRID: SCR_010761; version 1.1.0.46) with following options: ‐‐min‐mapping‐quality 20 ‐‐min‐base‐quality 20 ‐‐min‐coverage 5. Called SNVs and short indels were then annotated by SnpEff (RRID: SCR_005191; version 5.4.0a). Predicted off‐target sites were defined as loci in marmoset genome with perfect matches to the 3′‐terminal 8 bp of the gRNA target sequence, as well as the protospacer adjacent motif (PAM) sequence (NGG). SNVs and short indels overlapping the predicted off‐target sites were determined using BEDTools (RRID: SCR_006646; v2.30.0).

### 2.9 Histone Methylation Analysis

For histone methylation analysis, NaCl (cat. no. 198‐01675), digitonin (cat. no. 044‐02121), EDTA (cat. no. 345–01865), BSA (cat. no. 013‐15143), MgCl_2_ (cat. no. 132‐00175), SDS (cat. no. 191‐07145), ethanol (cat. no. 058‐00486) were purchased from FUJIFILM Wako. CUT&Tag was performed following previously described protocols (PMID: 31036827) using three fibroblast cultures at passage four (P4) independently expanded from the same P2 fibroblast population (regarded as three technical replicates). First, Concanavalin A beads (Bangs Laboratories, cat. no. BP531) were equilibrated in wash buffer containing 150 mM NaCl, 0.5 mM spermidine (Nacalai Tesque, cat. no. 32110‐41), protease inhibitor (Roche, cat. no. 11836145001), and 20 mM HEPES (Sigma‐Aldrich, cat. no. H0887; pH 7.5), and were subsequently bound to the cells. The cell‐bead complexes were then resuspended in primary antibody buffer (150 mM NaCl, 0.5 mM spermidine, protease inhibitor, 0.05% digitonin, 2 mM EDTA, 0.1% BSA, and 20 mM HEPES, pH 7.5) containing the target primary antibody (anti‐H3K4me3, Abcam, RRID: AB_306649; 1:100 dilution) and rotated overnight (16 h) at 4°C. Following washes with Dig‐wash buffer (150 mM NaCl, 0.5 mM spermidine, protease inhibitor, 0.05% digitonin, and 20 mM HEPES, pH 7.5), samples were incubated with a secondary antibody (Rockland, RRID: AB_218583) diluted in Dig‐wash buffer for 30 min at 24°C. After additional washes with Dig‐wash buffer, the cells were incubated with the pAG‐Tn5 adaptor complex for 1 h at 24°C. This was followed by washing with Dig‐300 buffer (300 mM NaCl, 0.5 mM spermidine, protease inhibitor, 0.01% digitonin, and 20 mM HEPES, pH 7.5) and tagmentation activation via incubation in Dig‐300 buffer supplemented with 10 mM MgCl_2_ for 1 h at 37°C. To halt the tagmentation reaction, 17 mM EDTA was added. DNA was then eluted using 0.1% SDS, and crosslinks were reversed by heating the samples at 65°C for 16 h. Samples were subsequently digested with 0.17 mg/mL Proteinase K (Nacalai Tesque, cat. no. 29442‐14) for 30 min at 50°C. DNA was purified via phenol–chloroform–isoamyl alcohol extraction followed by ethanol precipitation, washed with 70% ethanol and resuspended in water. Library amplification was performed using Q5 Hot Start High‐Fidelity 2× Master Mix (New England Biolabs, cat. no. M0494S) under the following thermal cycling conditions: 5 min at 72°C and 30 s at 98°C; followed by 12 cycles of 10 s at 98°C and 20 s at 63°C; and a final extension of 1 min at 72°C. Post‐PCR purification of the amplified DNA was conducted using solid‐phase reversible immobilization (SPRI) beads (Beckman Coulter, cat. no. B23318). The resulting libraries were sequenced on an Illumina NextSeq 2000 platform to generate 36‐bp paired‐end reads, yielding approximately 20 million reads per sample.

For downstream bioinformatics analysis, reads were mapped to the marmoset reference genome (calJac3) utilizing Bowtie2, and PCR duplicates were removed with the SAMtools (RRID: SCR_002105) rmdup function. For assessing peak distributions, H3K4me3 peaks were identified using MACS2 (RRID: SCR_013291) and high‐quality peaks with Irreproducible Discovery Rate (IDR; RRID: SCR_017237) < 0.05 were used. For assessing H3K4me3 deposition at promoters, read counts at promoter regions—defined as 1 kb upstream to 0.5 kb downstream of the TSS—were quantified by BAMscale. Finally, statistically significant differentially enriched regions were identified using the edgeR package (RRID: SCR_012802).

### 2.10 Transcriptome Analysis

For transcriptomic analysis, RNA‐seq was performed using three P4 fibroblast cultures independently expanded from the same P2 fibroblast population (regarded as three technical replicates). RNA‐seq libraries were generated using the SMART‐Seq Stranded Kit (Takara, cat. no. 634444). These libraries were then sequenced on an Illumina NextSeq 2000 platform to produce 36‐bp paired‐end reads, yielding an average of approximately 20 million reads per sample. Prior to alignment, adaptor sequences were trimmed utilizing Cutadapt (RRID: SCR_011841). The processed reads were subsequently aligned to the marmoset reference genome (calJac3) with HISAT2 (RRID: SCR_015530). To quantify gene expression, uniquely mapped reads were assigned to Ensembl gene models using featureCounts (RRID: SCR_012919). Statistical significance and false discovery rate (FDR) thresholds for differential expression were calculated using the edgeR package.

### 2.11 Quantification and Statistical Analysis

Because only a single *SETD1A* F1 mutant marmoset was available for analysis, no statistical method was employed to predetermine the sample sizes (biological replication = 1). Statistical analyses were performed exclusively on triplicate technical measurements obtained from the same animal and should therefore be interpreted as reflecting technical rather than biological variation. No tests for normality or outlier detection were performed. Statistical analyses were performed using GraphPad Prism 7 (RRID: SCR_000306), and the details of each test are specified in the figure legends. Statistical significance was defined as *p* < 0.05. Significance levels are indicated as follows: *p* < 0.05 (*), *p* < 0.001 (***), and not significant (ns).

## 3 Results

### 3.1 Generation of *SETD1A* Mutant Marmosets

**FIGURE 1:**
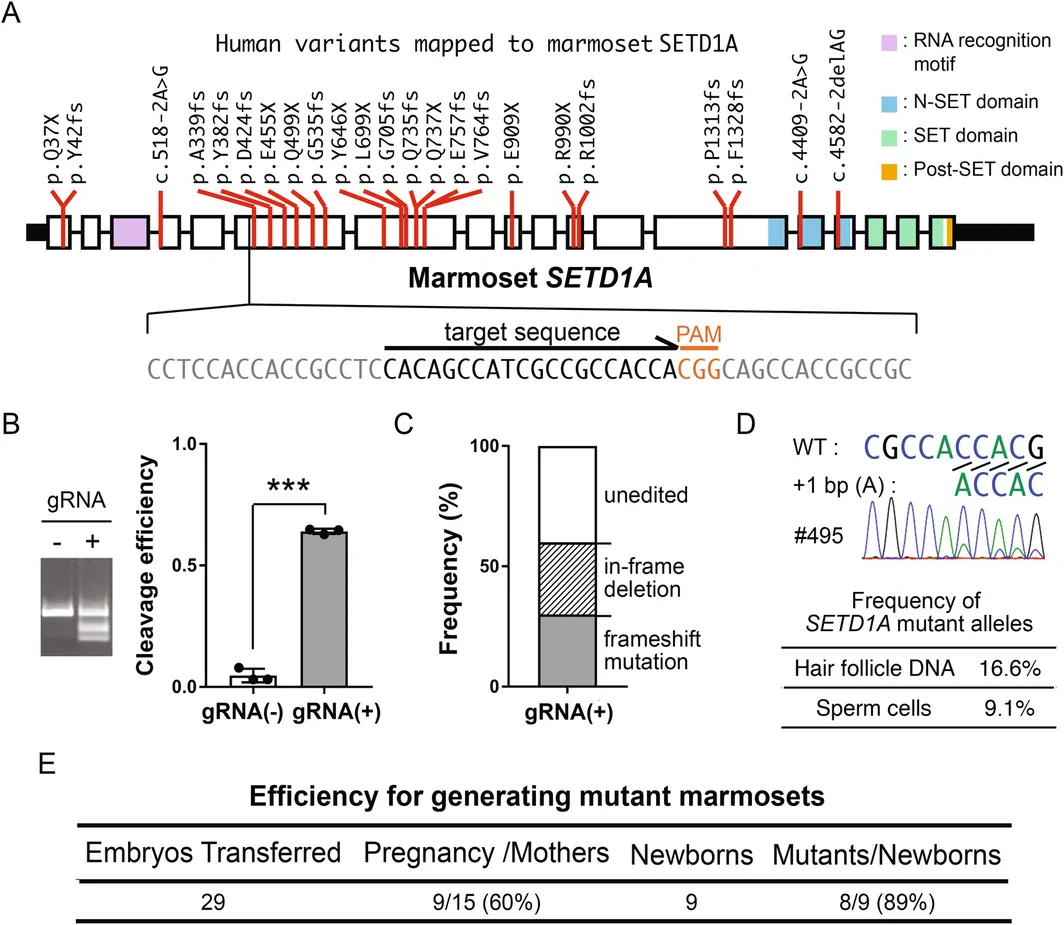
Generation of *SETD1A* mutant marmosets. (A) Schematic representation of the marmoset *SETD1A* gene. Squares indicate exons, and functional domains are color‐coded. Positions corresponding to loss‐of‐function variants identified in human patients are indicated above the gene. The target sequence of the designed guide RNA (gRNA) and the protospacer adjacent motif (PAM) sequence are shown below in bold and orange, respectively. (B) In vitro digestion assay using Cas9 and the gRNA. Left, representative agarose gel electrophoresis image of Cas9‐digested DNA fragments in the absence or presence of gRNA. Right, quantified cleavage efficiency (*n* = 3 digestion reactions for each condition; two‐tailed Welch's *t*‐test, *t*(2.6) = 35, *p* = 0.0002). Error bars indicate standard deviations (SD). (C) Allele frequencies of each mutation type in marmoset ES cells treated with the CRISPR/Cas9 system (*n* = 10 cloned PCR products; unedited = 4, in‐frame deletions = 3, frameshift mutations = 3). (D) Representative genotyping results of the founder mutant marmosets. Upper, Sanger sequencing chromatogram of hair follicle DNA from #495. Lower, frequencies of the mutant allele in hair follicle DNA, determined by amplicon sequencing, and in sperm DNA, determined by direct sequencing (*n* = 11 sperm cells; wild‐type sperm cells = 10, mutant sperm cell = 1) from #495. (E) Efficiency for mutant marmoset generation.

To investigate the impact of nonsense and frameshift mutations of *SETD1A* identified in human patients, we used the CRISPR/Cas9 system to engineer the marmoset *SETD1A* gene. Because many of these mutations are identified in exons 6 and 7, we designed a guide RNA (gRNA) targeting the 5′ side of exon 6 (Figure 1A). The mutagenesis efficiency of the gRNA was validated both in vitro and in marmoset ES cells (Figure 1B,C), and mutations were introduced at a frequency of approximately 60% in both assays. Then we adopted a method combining autologous embryo transfer with the CRISPR/Cas9 system to generate *SETD1A* mutant marmosets and obtained nine newborns following embryo manipulation. Two of the newborns died at delivery, possibly due to breech presentation. The newborns were genotyped by direct sequencing to determine the mutation types, and the allele frequencies were quantified by amplicon sequencing combined with CRISPResso2 analysis (Figure 1D; Table 1). Eight out of the nine newborns carried *SETD1A* mutations, and mutant allele frequencies substantially varied among individuals. Among the *SETD1A* mutant marmosets, three (#495, #498, and #502) carried the same frameshift mutation (1‐bp insertion of adenosine). The remaining animals carried only in‐frame mutations, most of which were 21‐bp deletions. In total, we manipulated 29 embryos from 15 female marmosets and nine were pregnant. The pregnancy rate (9 in 15 females; 60%) and mutagenesis rate (eight in nine newborns; 89%) were comparable to those we reported in a previous study generating *FMR1* mutant marmosets (Abe et al. 2021; Figure 1E).

**TABLE 1:** *SETD1A* founder mutants generated in this study.

No.	Sex	*SETD1A* mutation	Indel (bp)	Allele %	Note
#491	M	c.987_1007del; p.Thr330_Ala337del	−21	57.8 (hair follicle)	—
		wild type	—	42.2 (hair follicle)	
#492	F	c.970_990del; p.Ser324_Thr330del	−21	64.0 (hair follicle)	—
		c.991delinsTCCT; p.Thr330delinsSerSer	−3	36.0 (hair follicle)	
#495	M	wild type	—	83.4 (hair follicle)	—
		c.988_989insA; p.Thr330Asnfs*31	+1 (A)	16.6 (hair follicle)	
#497	M	wild type	—	89.4 (auricle) /82.0 (cortex)	died at delivery
		c.987_1007del; p.Thr330_Ala337del	−21	10.6 (auricle)/18.0 (cortex)	
#498	M	c.987_1007del; p.Thr330_Ala337del	−21	54.3 (auricle)/43.4 (cortex)	died at delivery
		c.988_989insA; p.Thr330Asnfs*31	+1 (A)	40.4 (auricle)/56.1 (cortex)	
		wild type	—	5.3 (auricle)/0.5 (cortex)	
#499	F	wild type	—	92.0 (hair follicle)	—
		c.980_1000del; p.Ile327_Ala333del	−21	8.0 (hair follicle)	
#500	F	wild type	—	82.8 (hair follicle)	—
		c.980_1000del; p.Ile327_Ala333del	−21	17.2 (hair follicle)	
#502	M	wild type	—	85.9 (hair follicle)	—
		c.988_989insA; p.Thr330Asnfs*31	+1 (A)	7.3 (hair follicle)	
		c.987_1007del; p.Thr330_Ala337del	−21	6.8 (hair follicle)	

*Note:* Genotypes of the eight mutant newborns are summarized. For genotyping, hair follicles were collected from the founder mutants, whereas auricle and cortical tissues were collected from the two individuals that died at delivery. Allele frequencies were determined by amplicon sequencing and CRISPResso2 analysis. Alleles with frequencies below 3% were regarded as sequencing errors and were excluded from calculation of allele percentages.

**FIGURE 2:**
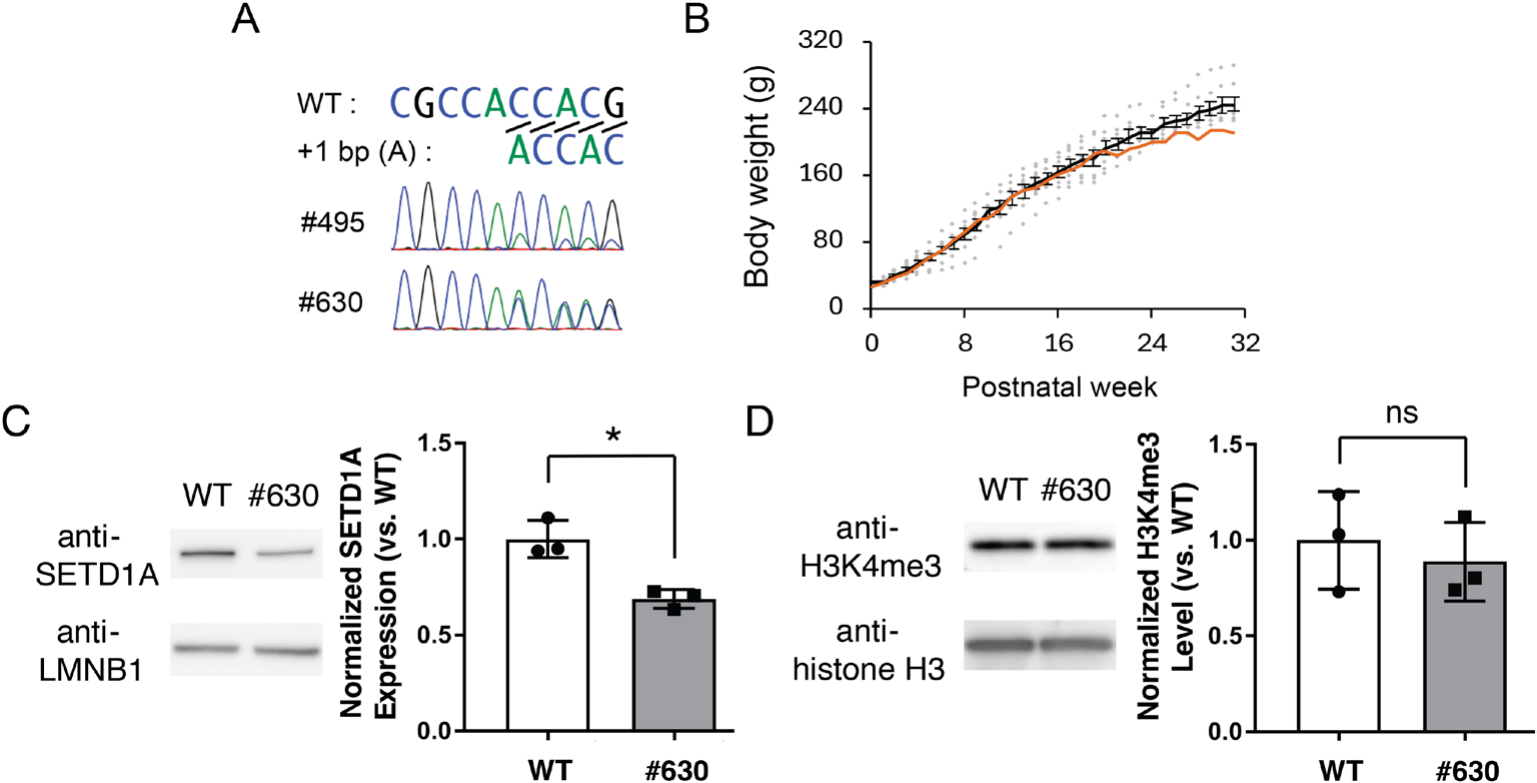
Evaluation of the *SETD1A* F1 mutant. (A) Sanger sequencing chromatograms of the *SETD1A* locus from #495 and #630. (B) Body weight gain curves during the first 31 postnatal weeks (black: Wild‐type, *n* = 8 animals; orange: #630). Each gray dot represents the body weight of one wild‐type individual. Error bars indicate standard error of the mean (SEM). (C) SETD1A protein levels in primary fibroblasts derived from the tails of one wild‐type marmoset and the SETD1A F1 mutant #630. Left, representative Western blot image. Right, quantitation of SETD1A protein levels normalized to LMNB1 (*n* = 3 replicate lanes from whole protein sample extracted from the same culture dishes; two‐tailed Welch's *t*‐test, *t* (3.0) = 5.0, *p* = 0.016). Error bars indicate SD. (D) Total histone H3 lysine‐4 trimethylation (H3K4me3) levels in primary fibroblasts derived from the tails of the wild‐type marmoset and #630. Left, representative Western blot image. Right, quantitation of H3K4me3 levels normalized to histone H3 (*n* = 3 replicate lanes from histone sample extracted from the same culture dishes; two‐tailed Welch's *t*‐test, *t* (3.8) = 0.59, *p* = 0.59). Error bars indicate SD. For (C) and (D), the replicate lanes represent technical replicates derived from the same biological sample. Accordingly, the reported *p*‐values reflect variation among technical replicates and should not be interpreted as evidence of biological significance.

Because mosaicism was observed in somatic cells of the *SETD1A* mutant marmosets, we next assessed the germline transmission. First, we isolated and genotyped sperm cells from #495, the male *SETD1A* mutant marmoset showing the highest allelic frequency of the *SETD1A* frameshift mutation. The proportion of mutant sperm cells was 9.1%, which was not markedly different from that estimated from its hair follicles (Figure 1D). We then mated #495 with wild‐type females and obtained an F1 mutant (#630) that inherited the frameshift mutation (Figure 2A). Because only a single *SETD1A* mutant marmoset was available for analysis, no statistical comparison was performed. The body weight trajectory of the mutant animal appeared comparable to those of male wild‐type marmosets (Figure 2B). Because genome editing using the CRISPR/Cas9 system can result in unintended mutations at other genomic loci, we performed whole‐genome sequencing for #630 and examined all 1882 potential off‐target sites, defined as loci with perfect matches to the PAM sequence and the eight nucleotides proximal to the PAM. No variants overlapping protein‐coding regions were detected at these sites.

### 3.2 Comparison of SETD1A Protein Expression and Total H3K4me3 Levels Between Fibroblasts From the *SETD1A* F1 Mutant and a Wild‐Type Marmoset

To assess SETD1A protein levels in the *SETD1A* F1 mutant #630, we performed Western blot analysis using fibroblasts derived from the tail biopsies of the mutant and one wild‐type marmoset (refer to Table S1 for information on the utilized marmosets). The SETD1A protein level normalized to LMNB1 in fibroblasts from #630 was 69% of that observed in fibroblasts from the wild‐type marmoset (Figure 2C; Figure S1A). Considering that SETD1A functions as a histone methyltransferase responsible for H3K4me3 deposition, we also assessed total H3K4me3 levels in fibroblasts from the two animals. We purified histone proteins and compared the fraction of trimethylated histone H3 between #630 and the wild‐type marmoset. The total H3K4me3 level in #630 fibroblasts was comparable to that observed in fibroblasts from the wild‐type marmoset (Figure 2D; Figure S1B).

### 3.3 Comparison of Epigenetic and Transcriptomic Profiles Between Fibroblasts From the *SETD1A* F1 Mutant and a Wild‐Type Marmoset

**FIGURE 3:**
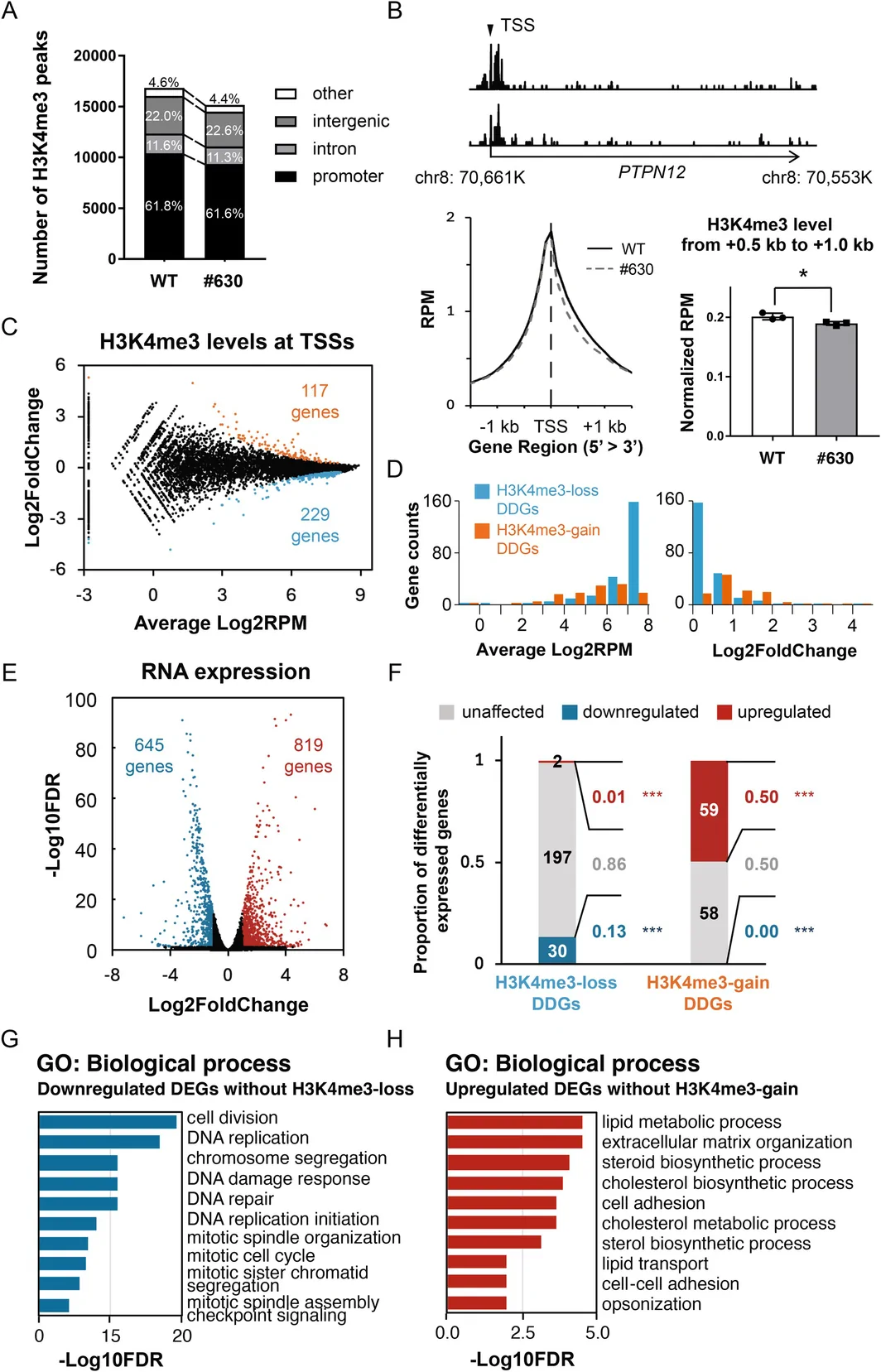
Epigenetic and transcriptomic profiling of fibroblasts derived from *SETD1A* F1 mutant. CUT&Tag and RNA‐seq analyses were performed using fibroblasts derived from one wild‐type marmoset and the *SETD1A* F1 mutant #630, with three technical replicates for each animal. Accordingly, the reported statistical measures, including *p*‐values and FDR values, reflect variation among technical replicates and should not be interpreted as evidence of biological significance between wild‐type and mutant marmosets. (A) Total number of detected histone H3 lysine‐4 trimethylation (H3K4me3) peaks and their proportion overlapping each genomic feature. (B) H3K4me3 peak profiles at transcription start sites (TSSs). Top, representative CUT&Tag signal at the *PTPN12* locus, a gene whose mouse homolog exhibits enrichment of H3K4me3 at its promoter in fibroblasts (ChIP‐Atlas; Oki et al. 2018). Bottom left, read counts per million mapped reads (RPM) from −1000 to +1000 bp relative to TSSs, averaged across genes and replicates. Bottom right, cumulative RPM within the region from +500 to +1000 bp downstream of TSSs normalized by cumulative RPM from −1000 to +1000 bp relative to TSSs (*n* = 3 technical replicates; two‐tailed Welch's *t*‐test, *t*(3.1) = 3.2, *p* = 0.047). Error bars indicate SD. (C) MA plot of genes with differential H3K4me3 deposition (DDGs) between fibroblasts from the mutant and wild‐type marmosets. RPM values from −1000 to +500 bp relative to TSSs were used for the analysis. DDGs were defined as false discovery rate (FDR) < 0.05. A total of 117 genes with increased H3K4me3 deposition are plotted in orange, and 229 genes with decreased H3K4me3 deposition are plotted in cyan. (D) Histograms showing the distributions of mean H3K4me3 enrichment levels (left) and fold changes (right) in DDGs. H3K4me3‐gain genes are shown in orange, whereas H3K4me3‐loss genes are shown in blue. (E) Volcano plot of differentially expressed genes (DEGs; *n* = 3 technical replicates). DEGs were defined as FDR < 0.05 and fold change ≥ 2 or ≤ 0.5. Eight hundred nineteen upregulated DEGs are plotted in red, and 645 downregulated DEGs are plotted in blue. (F) Enrichment and depletion analysis of DEGs among DDGs. The number and proportion of genes in each group are indicated. Among H3K4me3‐loss DDGs, downregulated DEGs were enriched, whereas upregulated DEGs were depleted. Conversely, among H3K4me3‐gain DDGs, upregulated DEGs were enriched, whereas downregulated DEGs were depleted. (***: *p* < 0.001; Fisher's exact test). (G) Gene Ontology (GO) terms for biological processes enriched among downregulated DEGs without decreased H3K4me3 deposition. The 10 terms with lowest FDR values are shown. (H) GO terms for biological processes enriched among upregulated DEGs without increased H3K4me3 deposition. The 10 terms with lowest FDR values are shown.

Because impaired histone methylation has been reported to alter the local H3K4me3 distribution without affecting the global H3K4me3 level, we performed CUT&Tag analysis to investigate the H3K4me3 deposition at individual genomic loci in fibroblasts derived from the *SETD1A* F1 mutant #630 and one wild‐type marmoset. We identified more than 15 000 H3K4me3 peaks across the marmoset genome. Genomic regions were classified into four categories (promoter, intron, intergenic, and other) as previously described (Mukai et al. 2019), and the overall distribution of H3K4me3 peaks across these regions was highly similar between the *SETD1A* F1 mutant #630 and a wild‐type marmoset, with approximately 62% of the peaks localized to promoter regions (defined as regions within 1.0 kb of TSSs; Figure 3A). When averaged across genes, the H3K4me3 signal exhibited a sharp peak centered at TSSs. The signal within the region from +0.5 to +1.0 kb downstream of TSSs was lower in #630 than in the wild‐type marmoset, whereas no apparent difference was observed upstream of TSSs (Figure 3B). Based on the analysis of technical replicates, 346 genes were identified as showing differential H3K4me3 deposition (DDGs) between #630 and the wild‐type marmoset (Figures 3C and S2). Among these, H3K4me3‐loss DDGs (229 genes) were approximately twice as frequent as H3K4me3‐gain DDGs (117 genes), and many of these H3K4me3‐loss DDGs exhibited high baseline H3K4me3 levels with relatively small differences between the two animals (Figure 3D). We then conducted an RNA‐seq analysis to assess the downstream transcriptional profiles of fibroblasts from #630 and the wild‐type marmoset (Figure 3E; Figure S2). Based on the analysis of technical replicates, 1464 differentially expressed genes (DEGs) were identified, including 645 downregulated DEGs and 819 upregulated DEGs. Downregulated and upregulated DEGs were enriched among H3K4me3‐loss and H3K4me3‐gain DDGs, respectively, whereas the opposite combinations were depleted (Figure 3F). However, most DEGs were not accompanied by differential H3K4me3 deposition at their TSSs. Therefore, we performed enrichment analyses focusing on DEGs without differential H3K4me3 deposition. Downregulated DEGs without lower H3K4me3 deposition included genes related to processes such as mitotic cell division and DNA repair (Figure 3G; Table S3), whereas upregulated DEGs without higher H3K4me3 deposition included genes related to processes such as lipid metabolism and cell adhesion (Figure 3H; Table S4).

## 4 Discussion

In this study, we established a novel *SETD1A* mutant marmoset lineage which is, to the best of our knowledge, the first genetically engineered marmoset carrying a mutation in an epigenetic regulatory gene associated with SCZ. We introduced a frameshift mutation into the marmoset *SETD1A* gene and confirmed its germline transmission to the next generation (Figures 1 and 2A). In fibroblasts derived from the *SETD1A* F1 mutant #630, a lower SETD1A protein level, lower H3K4me3 deposition downstream of TSSs, and differences in transcriptomic profiles were observed compared with fibroblasts derived from a wild‐type marmoset (Figures 2C and 3B,E). These observations are consistent with the known regulatory functions of SETD1A.

### 4.1 Efficiency of Mutant Animal Generation and Distribution of Mutant Types

Using autologous embryo transfer combined with the CRISPR/Cas9 system, we successfully generated mutant marmosets with pregnancy and mutagenesis rates comparable to those reported in previous studies (Abe et al. 2021; Figure 1E), further supporting the applicability of this method to different genomic loci in the marmoset genome. However, a substantial bias was observed in the mutation types introduced to the *SETD1A* mutant marmosets. Among the 11 mutant alleles identified, seven consisted of three types of 21‐bp deletions, whereas three were the same 1‐bp insertion of adenosine. This bias is likely attributable to the presence of two 5′‐CAGCCAYCGCCGC‐3′ sequences flanking the CRISPR/Cas9 cleavage site at a 21‐bp interval, which may facilitate repair through the microhomology‐mediated end joining (MMEJ) pathway. It is noteworthy that several computational tools predicted this bias. For example, inDelphi (Shen et al. 2018) predicted the 1‐bp insertion of adenosine as the second most probable repair outcome. In addition, Microhomology‐predictor, a tool that evaluates the likelihood of in‐frame mutations introduced via the MMEJ pathway (Bae et al. 2014), produced a low out‐of‐frame score for our target site. Therefore, using computational prediction for CRISPR/Cas9 repair outcomes could be useful in selecting target sites when generating mutant marmosets.

### 4.2 Lower SETD1A Protein and mRNA Levels in the *SETD1A* F1 Mutant

The SETD1A protein level in fibroblasts derived from the *SETD1A* F1 mutant #630 was 69% of that observed in fibroblasts derived from a wild‐type marmoset (Figure 2C). Consistently, RNA‐seq analysis revealed that the *SETD1A* mRNA level in #630 fibroblasts was also 69% of that in the wild‐type fibroblasts (log2 fold change = −0.54 according to edgeR analysis). In addition, no RNA‐seq reads containing the *SETD1A* mutation (a 1‐bp insertion of adenosine) were detected in any of the three technical replicates from #630 fibroblasts. This observation is consistent with the possibility that transcripts from the mutant *SETD1A* allele undergo nonsense‐mediated decay. Thus, the lower SETD1A protein and mRNA levels observed in #630, together with the absence of detectable transcripts from the mutant allele, are consistent with an effect of the frameshift mutation on *SETD1A* expression. However, because these molecular analyses were based on fibroblasts from only one mutant and one wild‐type animal, a contribution of inter‐individual variation cannot be excluded.

### 4.3 Epigenomic and Transcriptomic Characterization of *SETD1A* Mutant Fibroblasts

#### 4.3.1 Global Levels and Distribution of H3K4me3 Were Largely Similar Between the Mutant and Wild‐Type Marmosets

Despite the known histone methyltransferase activity of SETD1A, H3K4me3 deposition patterns were largely similar between the *SETD1A* F1 mutant #630 and a wild‐type marmoset. No apparent difference was observed in global H3K4me3 level or in its overall genomic distribution, and even when focusing on genomic regions surrounding promoters, only a modest difference, with lower H3K4me3 deposition in #630, was detected downstream of TSSs. Limited effects of *SETD1A* heterozygous mutations on H3K4me3 deposition have been reported in different experimental contexts. In mouse ES cells, conditional knockout of a single *Setd1a* allele (Bledau et al. 2014) or complete elimination of the catalytic domain of SETD1A (Sze et al. 2017) had little effect on H3K4me3 levels. Furthermore, in human forebrain organoids harboring a heterozygous loss‐of‐function mutation in *SETD1A*, no widespread alternation of SETD1A binding sites or H3K4me3 peaks was identified (Sun et al. 2025). These reports suggest that the effects of heterozygous *SETD1A* mutations on epigenomic regulation may be restricted to specific genomic loci rather than causing global alterations. The relatively limited differences observed between #630 and the wild‐type marmoset are consistent with this possibility.

One possible explanation underlying the limited effects of SETD1A deficiency on H3K4me3 deposition is compensation by other KMT2 family members such as MLL2 and SETD1B, which also catalyze H3K4me3 deposition. Indeed, the suppression of *SETD1A* expression or truncation of its catalytic domain has been reported to induce compensatory activity by these related methyltransferases in rodents (Sze et al. 2020; Zhang et al. 2025). Notably, in the cortex of mice with conditional *Setd1b* knockout in excitatory neurons, reduced H3K4me3 deposition downstream of TSSs was observed preferentially in genes characterized by broad H3K4me3 peaks at TSSs. This pattern is similar to that observed in our comparison of #630 and the wild‐type marmoset (Figure 3B–D), suggesting that SETD1A and SETD1B may share similar regulatory mechanisms governing H3K4me3 deposition. However, additional biological replicates will be required to determine whether the H3K4me3 differences observed in #630 are attributable to the *SETD1A* mutation.

#### 4.3.2 Differences in Transcriptomic Profiles Between the Mutant and Wild‐Type Marmosets

In contrast to the relatively modest differences in H3K4me3 deposition, more extensive differences between transcriptomic profiles were observed between the *SETD1A* F1 mutant #630 and the wild‐type marmoset. Among genes identified as DDGs and DEGs based on technical replicates, H3K4me3‐loss DDGs were positively associated with downregulated DEGs, whereas H3K4me3‐gain DDGs were positively associated with upregulated DEGs (Figure 3F). This association is consistent with a relationship between differences in H3K4me3 deposition and gene expression. However, genes showing differential H3K4me3 deposition only account for a small proportion of the DEGs (30 H3K4me3‐loss DDGs among 645 downregulated DEGs [4.7%] and 59 H3K4me3‐gain DDGs among 819 upregulated DEGs [7.2%]; Figure 3E,F). Several factors may account for the remaining differences in gene expression. First, as discussed below, inter‐individual variation cannot be distinguished from effects associated with the *SETD1A* mutation in the present analysis. Second, secondary transcriptional effects may contribute, where DEGs with differential H3K4me3 deposition influence the expression of downstream target genes. In addition, SETD1A is known to exert noncanonical functions independently of its catalytic domain. In mouse ES cells, SETD1A is required for cell survival and proliferation whereas a truncated form lacking the catalytic domain is sufficient for these functions (Sze et al. 2017). Furthermore, a functional domain upstream of the catalytic domain has been reported to be critical for regulating cell proliferation and DNA damage responses (Hoshii 2025). Genes related to cell division and DNA repair were enriched among downregulated DEGs without decreased H3K4me3 deposition (Figure 3G). Although this observation is consistent with previously reported noncanonical functions of *SETD1A*, additional biological replicates are required to establish whether these transcriptional differences are attributable to *SETD1A* deficiency. Moreover, these observations were obtained in proliferating fibroblasts and may therefore reflect cell‐type‐specific processes. Their relevance to the neuropsychiatric disorders associated with *SETD1A* remains to be determined in future studies using appropriate disease‐relevant tissues.

### 4.4 Limitations

This study has several limitations. First, because only one *SETD1A* F1 mutant and one wild‐type marmoset were used for the molecular analyses, the observed differences in SETD1A protein levels, H3K4me3 deposition, and transcriptomic profiles cannot be attributed specifically to the *SETD1A* mutation and may, at least in part, reflect inter‐individual differences in genetic background or other biological factors. Moreover, the three replicates used for the CUT&Tag and RNA‐seq analyses were technical replicates derived from the same fibroblast population rather than independent biological replicates. Therefore, the statistical measures obtained from these analyses reflect technical variation and cannot establish biological significance between mutant and wild‐type marmosets. Additional mutant and wild‐type animals will be necessary to determine which of the observed molecular differences are reproducibly associated with the *SETD1A* mutation. Second, we used fibroblasts for molecular analyses but in *SETD1A*‐associated diseases, such as SCZ, the effects of *SETD1A* dysfunction in the central nervous system should be evaluated. Therefore, further studies involving larger cohorts of animals and analyses of disease‐relevant tissues will be required.

## 5 Conclusion

Non‐human primate models that mimic SCZ have served as valuable tools for investigating disease mechanisms and developing therapeutic drugs, although many such models have traditionally relied on pharmacological manipulations, including the administration of psychotomimetic drugs (reviewed in Morris et al. 2005). In this study, we established, to the best of our knowledge, the first genetically engineered non‐human primate lineage carrying a mutation in a genetic risk factor for SCZ and demonstrated germline transmission of the mutant allele. Although further studies with additional animals are required to establish the molecular and phenotypic consequences of the *SETD1A* mutation, this marmoset lineage provides a genetic platform for future studies of the effects of *SETD1A* deficiency in a non‐human primate. In particular, characterization of this lineage may provide opportunities to investigate cognitive, molecular, and neurobiological phenotypes associated with *SETD1A* deficiency across development.

## Author Contributions

**Harumi Nakao:** writing – review and editing, formal analysis, investigation. **Yuichi Hiraoka:** writing – review and editing, funding acquisition. **Atsu Aiba:** writing – review and editing, conceptualization, funding acquisition, supervision. **Motoki Goto:** resources. **Moe Tamano:** formal analysis. **Yukiko Abe:** resources. **Maria Harbers:** writing – review and editing, resources. **Mai Saeki:** formal analysis, investigation. **Yusuke Kishi:** writing – original draft, methodology, formal analysis, funding acquisition. **Zefeng Wei:** writing – original draft, conceptualization, methodology, formal analysis, investigation.

## Funding

This work is supported by Brain/MINDS (JP19dm0207071 to A.A.) and Brain/MINDS 2.0 (JP23wm0625001 to A.A.) from AMED, Grants‐in‐Aid for Scientific Research from JSPS (Number 24K02020 to Y.K., Number 23K05974 to Y.H.), the Ono Pharmaceutical Foundation for Oncology, Immunology, and Neurology to Y.K., and Takahashi Industrial and Economic Research Foundation to Y.K.

## Conflicts of Interest

The authors declare no conflicts of interest.

## Supporting information


**Figure S1:** The whole membrane images of the Western blot analyses.
**Figure S2:** Principal component analysis (PCA) of the CUT&Tag and RNA‐seq datasets.
**Table S1:** Information of the *SETD1A* F1 mutant #630 and all used wild‐type marmosets.
**Table S2:** Details of embryo manipulation for each *SETD1A* F0 mutant.
**Table S3:** Gene Ontology (GO) terms associated with downregulated DEGs without H3K4me3‐loss.
**Table S4:** Gene Ontology (GO) terms associated with upregulated DEGs without H3K4me3‐gain.

## Data Availability

Raw sequence data of RNA‐seq, CUT&Tag, and whole genome sequencing presented in this study have been deposited in the NCBI BioProject database under accession number PRJNA1475781. Results of EdgeR analysis for RNA‐seq and CUT&Tag have been deposited in the Gene Expression Omnibus (GEO) database under accession number GSE334806. *GSE334806 is currently accessible via reviewer token “arepuiuyllmhzqj”.
