# Supplementary material for: Establishment of a Common Marmoset Lineage Carrying a Frameshift Mutation in SETD1A, a Schizophrenia Risk Gene
Source: J Neurochem. 2026 Sep 3;170(9):e70546. doi: 10.1111/jnc.70546 (PMC13539942; doi:10.1111/jnc.70546)
Supplement: Supplementary file 1 [file JNC-170-e70546-s001.zip › jnc70546-sup-0001-FigureS1-S2-TableS1-S4@SupplementaryInformation.pdf]

Supplementary Information

Establishment of a Common Marmoset Lineage Carrying a Frameshift Mutation in *SETD1A*, a Schizophrenia Risk Gene

Zefeng Wei[1], Harumi Nakao[1], Yuichi Hiraoka[1], Maria Harbers[1], Yukiko Abe[1], Motoki Goto[1], Moe Tamano[1], Mai Saeki[2], Yusuke Kishi[2,3,4], Atsu Aiba[1‡]

[1]Laboratory of Animal Resources, Center for Disease Biology and Integrative Medicine, Graduate School of Medicine, The University of Tokyo, Tokyo, Japan

[2]Laboratory of Molecular Neurobiology, Institute for Quantitative Biosciences, The University of Tokyo, Tokyo, Japan

[3]Laboratory of Molecular Biology, Graduate School of Pharmaceutical Sciences, The University of Tokyo, Tokyo, Japan

[4]Division of Aging Biology, Research Institute for Science and Technology, Tokyo University of Science, Chiba, Japan

‡Correspondence should be addressed to:

Atsu Aiba, aiba@m.u-tokyo.ac.jp, tel: +81-3-5841-3638

Supplementary Information Contents

Figure S1: The whole membrane images of the Western blot analyses.

Figure S2: Principal component analysis (PCA) of the CUT&Tag and RNA-seq datasets.

Table S1: Information of the SETD1A F1 mutant #630 and all used wild-type marmosets.

Table S2: Details of embryo manipulation for each *SETD1A* F0 mutant.

Table S3: Gene Ontology (GO) terms associated with downregulated DEGs without H3K4me3-loss.

Table S4: Gene Ontology (GO) terms associated with upregulated DEGs without H3K4me3-gain.

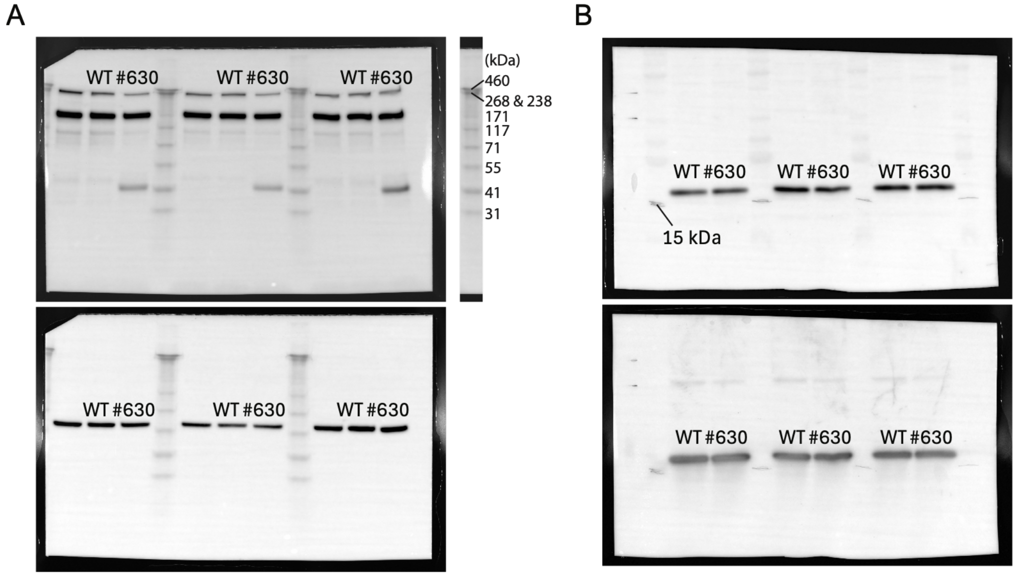

Figure S1 The whole membrane images of the Western blot analyses performed in this study are shown (WT: wild-type, #630: the *SETD1A* F1 mutant. n = 3 replicate lanes from whole protein sample extracted from the same culture dishes). Chemiluminescence images and colorimetric images were overlayed. (A) Immunoblotting for SETD1A (upper) and LMNB1 (lower). The HiMark™ Pre-stained Protein Standard (Thermo Fisher Scientific, cat. no. LC5699) was used as a molecular weight marker. (B) Immunoblotting for H3K4me3 (upper) and Histone H3 (lower). The BenchMark™ Protein Ladder (Thermo Fisher Scientific, cat. no. 10747012) was used as a molecular weight marker.

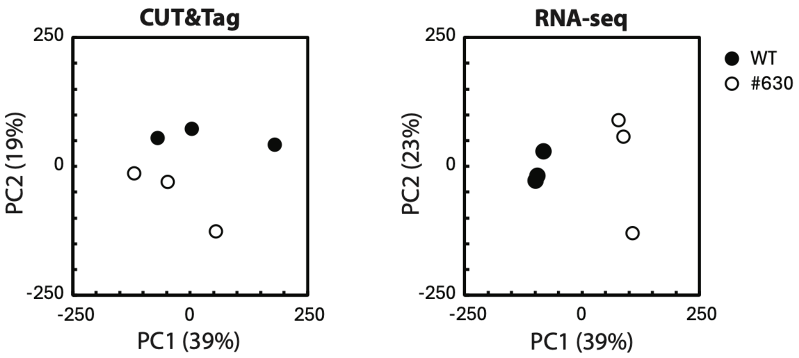

Figure S2 Principal component analysis (PCA) of the CUT&Tag (left) and RNA-seq (right) datasets generated in this study. The proportion of variance explained by each principal component is shown in the axis labels. Each data point represents a technical replicate (black: wild-type, white: the *SETD1A* F1 mutant (#630). n = 3 technical replicates).

Table S1 Information of the *SETD1A* F1 mutant #630 and all used wild-type marmosets.

No.	Parents (Dam × Sire)	Date of birth	Sex	Genotype	Usage	Tail sampling date
#630	#356 × #495	2025.08.20	male	*SETD1A* mutant	Western blot, CUT&Tag, RNA-seq body weight gain	2025.08.20 (postnatal day 0)
#311	#144 × #60	2020.07.04		wild type	Western blot, CUT&Tag, RNA-seq	2020.07.07 (postnatal day 3)
#568	#365 × #485	2024.06.02			body weight gain	-
#572	#442 × #491	2024.07.08				
#583	#402 × #286	2024.10.15				
#586	#157 × #286	2024.10.28				
#588	#351 × #485	2024.10.28				
#610	#240 × #485	2025.04.08				
#620	#346 × #495	2025.07.12				
#625	#500 × #410	2025.08.13				

Table S2 Details of embryo manipulation for each *SETD1A* F0 mutant.

No.	Injection Details	Cas9 protein (ng/μL)	crRNA (ng/μL)	tracrRNA (ng/μL)
#491	one injected into cytoplasm at pronulear stage,	100	50	50
#492	one injected into cytoplasm at one-cell stage			
#495	injected into one pronuleus at pronulear stage	50	25	25
#497	injected into one pronuleus at pronulear stage	50	25	25
#498	one injected into one pronucleus at pronulear stage,	50	25	25
#499	one injected into cytoplasm at one-cell stage,			
#500	one injected into one blastomere at two-cell stage			
#502	injected into one pronuleus at pronulear stage	50	25	25

Table S3 GO terms associated with downregulated DEGs without H3K4me3-loss.

Category	Term	Count	List Total	Pop Hits	Pop Total	P-Value	Benjamini	Fold Enrichment	Bonferroni	FDR	Fisher Exact
GOTERM_BP_DIRECT	cell division	71	567	433	19512	1.97E-32	5.14E-29	5.64	5.14E-29	5.03E-29	3.02E-33
GOTERM_BP_DIRECT	DNA replication	40	567	134	19512	2.06E-28	2.68E-25	10.27	5.36E-25	2.62E-25	1.46E-29
GOTERM_BP_DIRECT	chromosome segregation	30	567	112	19512	7.05E-20	6.12E-17	9.22	1.84E-16	5.98E-17	5.84E-21
GOTERM_BP_DIRECT	DNA repair	55	567	437	19512	1.35E-19	7.06E-17	4.33	3.53E-16	6.90E-17	2.82E-20
GOTERM_BP_DIRECT	DNA damage response	64	567	584	19512	1.24E-19	7.06E-17	3.77	3.23E-16	6.90E-17	3.02E-20
GOTERM_BP_DIRECT	DNA replication initiation	15	567	27	19512	3.63E-15	1.58E-12	19.12	9.54E-12	1.54E-12	9.14E-17
GOTERM_BP_DIRECT	mitotic spindle organization	18	567	58	19512	3.77E-13	1.40E-10	10.68	9.82E-10	1.37E-10	2.56E-14
GOTERM_BP_DIRECT	mitotic cell cycle	26	567	146	19512	7.67E-13	2.50E-10	6.13	2.00E-09	2.44E-10	1.06E-13
GOTERM_BP_DIRECT	mitotic sister chromatid segregation	14	567	38	19512	2.50E-11	7.24E-09	12.68	6.52E-08	7.08E-09	1.33E-12
GOTERM_BP_DIRECT	double-strand break repair via break-induced replication	8	567	12	19512	1.16E-08	2.75E-06	22.94	3.03E-05	2.69E-06	2.16E-10
GOTERM_BP_DIRECT	mitotic spindle assembly checkpoint signaling	11	567	30	19512	6.94E-09	1.81E-06	12.62	1.81E-05	1.77E-06	3.75E-10
GOTERM_BP_DIRECT	regulation of DNA-templated DNA replication initiation	8	567	14	19512	4.79E-08	9.59E-06	19.66	1.25E-04	9.38E-06	1.25E-09
GOTERM_BP_DIRECT	G1/S transition of mitotic cell cycle	16	567	88	19512	3.24E-08	7.04E-06	6.26	8.45E-05	6.88E-06	4.38E-09
GOTERM_BP_DIRECT	reciprocal meiotic recombination	10	567	33	19512	2.83E-07	5.26E-05	10.43	7.36E-04	5.14E-05	2.01E-08
GOTERM_BP_DIRECT	spindle organization	8	567	20	19512	9.29E-07	1.51E-04	13.77	2.42E-03	1.48E-04	4.48E-08
GOTERM_BP_DIRECT	positive regulation of chromosome condensation	6	567	9	19512	2.31E-06	3.16E-04	22.94	5.99E-03	3.09E-04	4.57E-08
GOTERM_BP_DIRECT	double-strand break repair via homologous recombination	17	567	123	19512	5.48E-07	9.51E-05	4.76	1.42E-03	9.29E-05	1.02E-07
GOTERM_BP_DIRECT	mitotic metaphase chromosome alignment	11	567	49	19512	1.16E-06	1.77E-04	7.73	3.01E-03	1.73E-04	1.21E-07
GOTERM_BP_DIRECT	replication fork processing	10	567	41	19512	2.09E-06	3.02E-04	8.39	5.42E-03	2.95E-04	1.98E-07
GOTERM_BP_DIRECT	positive regulation of chromosome separation	6	567	11	19512	8.07E-06	9.05E-04	18.77	2.08E-02	8.85E-04	2.39E-07
GOTERM_BP_DIRECT	protein localization to kinetochore	6	567	11	19512	8.07E-06	9.05E-04	18.77	2.08E-02	8.85E-04	2.39E-07
GOTERM_BP_DIRECT	chromosome organization	11	567	53	19512	2.47E-06	3.22E-04	7.14	6.42E-03	3.15E-04	2.85E-07
GOTERM_BP_DIRECT	G2/M transition of mitotic cell cycle	11	567	54	19512	2.96E-06	3.66E-04	7.01	7.67E-03	3.58E-04	3.49E-07

Category	Term	Count	List Total	Pop Hits	Pop Total	P-Value	Benjamini	Fold Enrichment	Bonferroni	FDR	Fisher Exact
GOTERM_BP_DIRECT	attachment of spindle microtubules to kinetochore	7	567	19	19512	1.14E-05	1.08E-03	12.68	2.93E-02	1.05E-03	6.27E-07
GOTERM_BP_DIRECT	mitotic G2 DNA damage checkpoint signaling	9	567	37	19512	8.77E-06	9.13E-04	8.37	2.26E-02	8.93E-04	8.34E-07
GOTERM_BP_DIRECT	chromosome condensation	8	567	28	19512	1.16E-05	1.08E-03	9.83	2.98E-02	1.05E-03	9.00E-07
GOTERM_BP_DIRECT	DNA-templated DNA replication	10	567	48	19512	8.34E-06	9.05E-04	7.17	2.15E-02	8.85E-04	9.60E-07
GOTERM_BP_DIRECT	DNA strand elongation involved in DNA replication	5	567	8	19512	4.47E-05	3.69E-03	21.51	1.10E-01	3.61E-03	1.06E-06
GOTERM_BP_DIRECT	positive regulation of chromosome segregation	6	567	14	19512	3.25E-05	2.92E-03	14.75	8.12E-02	2.85E-03	1.44E-06
GOTERM_BP_DIRECT	double-strand break repair	13	567	90	19512	1.10E-05	1.08E-03	4.97	2.83E-02	1.05E-03	1.95E-06
GOTERM_BP_DIRECT	nuclear division	5	567	9	19512	7.86E-05	5.85E-03	19.12	1.85E-01	5.72E-03	2.33E-06
GOTERM_BP_DIRECT	centriole replication	7	567	23	19512	3.84E-05	3.34E-03	10.47	9.52E-02	3.26E-03	2.76E-06
GOTERM_BP_DIRECT	homologous chromosome pairing at meiosis	8	567	34	19512	4.53E-05	3.69E-03	8.1	1.11E-01	3.61E-03	4.51E-06
GOTERM_BP_DIRECT	mitotic spindle assembly	9	567	46	19512	4.71E-05	3.72E-03	6.73	1.15E-01	3.63E-03	5.85E-06
GOTERM_BP_DIRECT	mitotic chromosome condensation	6	567	18	19512	1.26E-04	8.66E-03	11.47	2.80E-01	8.47E-03	8.08E-06
GOTERM_BP_DIRECT	positive regulation of mitotic cell cycle spindle assembly checkpoint	5	567	11	19512	1.97E-04	1.22E-02	15.64	4.01E-01	1.19E-02	8.14E-06
GOTERM_BP_DIRECT	mitotic spindle midzone assembly	5	567	11	19512	1.97E-04	1.22E-02	15.64	4.01E-01	1.19E-02	8.14E-06
GOTERM_BP_DIRECT	CENP-A containing chromatin assembly	5	567	11	19512	1.97E-04	1.22E-02	15.64	4.01E-01	1.19E-02	8.14E-06
GOTERM_BP_DIRECT	kinetochore organization	4	567	6	19512	4.55E-04	2.40E-02	22.94	6.94E-01	2.34E-02	1.01E-05
GOTERM_BP_DIRECT	negative regulation of meiotic nuclear division	4	567	6	19512	4.55E-04	2.40E-02	22.94	6.94E-01	2.34E-02	1.01E-05
GOTERM_BP_DIRECT	homologous recombination	5	567	12	19512	2.88E-04	1.70E-02	14.34	5.28E-01	1.67E-02	1.36E-05
GOTERM_BP_DIRECT	positive regulation of G2/M transition of mitotic cell cycle	7	567	29	19512	1.56E-04	1.04E-02	8.31	3.34E-01	1.02E-02	1.51E-05
GOTERM_BP_DIRECT	DNA recombination	14	567	125	19512	7.06E-05	5.41E-03	3.85	1.68E-01	5.29E-03	1.66E-05
GOTERM_BP_DIRECT	DNA replication checkpoint signaling	5	567	13	19512	4.07E-04	2.30E-02	13.24	6.53E-01	2.25E-02	2.16E-05

Category	Term	Count	List Total	Pop Hits	Pop Total	P-Value	Benjamini	Fold Enrichment	Bonferroni	FDR	Fisher Exact
GOTERM_BP_DIRECT	DNA replication-dependent chromatin assembly	4	567	7	19512	7.79E-04	3.56E-02	19.66	8.69E-01	3.48E-02	2.30E-05
GOTERM_BP_DIRECT	meiotic chromosome condensation	4	567	7	19512	7.79E-04	3.56E-02	19.66	8.69E-01	3.48E-02	2.30E-05
GOTERM_BP_DIRECT	meiotic chromosome segregation	4	567	7	19512	7.79E-04	3.56E-02	19.66	8.69E-01	3.48E-02	2.30E-05
GOTERM_BP_DIRECT	cellular response to leukemia inhibitory factor	13	567	113	19512	1.09E-04	7.66E-03	3.96	2.47E-01	7.49E-03	2.48E-05
GOTERM_BP_DIRECT	DNA strand resection involved in replication fork processing	5	567	14	19512	5.56E-04	2.84E-02	12.29	7.65E-01	2.78E-02	3.28E-05
GOTERM_BP_DIRECT	chromatin organization	30	567	462	19512	9.06E-05	6.55E-03	2.23	2.10E-01	6.40E-03	3.86E-05
GOTERM_BP_DIRECT	regulation of mitotic nuclear division	6	567	23	19512	4.40E-04	2.40E-02	8.98	6.82E-01	2.34E-02	3.88E-05
GOTERM_BP_DIRECT	microtubule-based movement	11	567	87	19512	2.10E-04	1.27E-02	4.35	4.21E-01	1.24E-02	4.30E-05
GOTERM_BP_DIRECT	regulation of DNA damage checkpoint	5	567	15	19512	7.41E-04	3.56E-02	11.47	8.55E-01	3.48E-02	4.80E-05
GOTERM_BP_DIRECT	DNA biosynthetic process	7	567	35	19512	4.60E-04	2.40E-02	6.88	6.98E-01	2.34E-02	5.59E-05
GOTERM_BP_DIRECT	metaphase chromosome alignment	5	567	16	19512	9.66E-04	4.12E-02	10.75	9.19E-01	4.03E-02	6.82E-05
GOTERM_BP_DIRECT	mitotic sister chromatid cohesion	5	567	16	19512	9.66E-04	4.12E-02	10.75	9.19E-01	4.03E-02	6.82E-05
GOTERM_BP_DIRECT	nuclear chromosome segregation	4	567	9	19512	1.79E-03	7.17E-02	15.29	9.91E-01	7.01E-02	7.91E-05
GOTERM_BP_DIRECT	positive regulation of DNA-directed DNA polymerase activity	4	567	9	19512	1.79E-03	7.17E-02	15.29	9.91E-01	7.01E-02	7.91E-05
GOTERM_BP_DIRECT	cellular response to cisplatin	3	567	4	19512	4.85E-03	1.62E-01	25.81	1.00E+00	1.58E-01	9.55E-05
GOTERM_BP_DIRECT	nucleocytoplasmic transport	8	567	52	19512	7.18E-04	3.56E-02	5.29	8.46E-01	3.48E-02	1.18E-04
GOTERM_BP_DIRECT	regulation of cell cycle	20	567	269	19512	3.44E-04	1.99E-02	2.56	5.92E-01	1.95E-02	1.26E-04
GOTERM_BP_DIRECT	positive regulation of DNA replication	6	567	29	19512	1.35E-03	5.56E-02	7.12	9.70E-01	5.44E-02	1.57E-04
GOTERM_BP_DIRECT	mitotic cytokinesis	9	567	69	19512	8.42E-04	3.73E-02	4.49	8.89E-01	3.64E-02	1.66E-04
GOTERM_BP_DIRECT	mitotic DNA replication checkpoint signaling	4	567	11	19512	3.37E-03	1.20E-01	12.51	1.00E+00	1.17E-01	1.98E-04
GOTERM_BP_DIRECT	error-prone translesion synthesis	4	567	11	19512	3.37E-03	1.20E-01	12.51	1.00E+00	1.17E-01	1.98E-04
GOTERM_BP_DIRECT	regulation of attachment of spindle microtubules to kinetochore	4	567	11	19512	3.37E-03	1.20E-01	12.51	1.00E+00	1.17E-01	1.98E-04
GOTERM_BP_DIRECT	sister chromatid cohesion	5	567	20	19512	2.34E-03	8.98E-02	8.6	9.98E-01	8.78E-02	2.20E-04
GOTERM_BP_DIRECT	mismatch repair	6	567	31	19512	1.84E-03	7.24E-02	6.66	9.92E-01	7.08E-02	2.32E-04

Category	Term	Count	List Total	Pop Hits	Pop Total	P-Value	Benjamini	Fold Enrichment	Bonferroni	FDR	Fisher Exact
GOTERM_BP_DIRECT	mitotic DNA replication initiation	3	567	5	19512	7.92E-03	2.27E-01	20.65	1.00E+00	2.22E-01	2.34E-04
GOTERM_BP_DIRECT	resolution of DNA recombination intermediates	3	567	5	19512	7.92E-03	2.27E-01	20.65	1.00E+00	2.22E-01	2.34E-04
GOTERM_BP_DIRECT	positive regulation of potassium ion import across plasma membrane	3	567	5	19512	7.92E-03	2.27E-01	20.65	1.00E+00	2.22E-01	2.34E-04
GOTERM_BP_DIRECT	meiotic cell cycle	13	567	141	19512	8.45E-04	3.73E-02	3.17	8.89E-01	3.64E-02	2.44E-04
GOTERM_BP_DIRECT	protein localization to chromatin	6	567	32	19512	2.12E-03	8.26E-02	6.45	9.96E-01	8.07E-02	2.79E-04
GOTERM_BP_DIRECT	positive regulation of apoptotic process	22	567	333	19512	7.72E-04	3.56E-02	2.27	8.66E-01	3.48E-02	3.18E-04
GOTERM_BP_DIRECT	mRNA transport	12	567	128	19512	1.27E-03	5.32E-02	3.23	9.63E-01	5.20E-02	3.59E-04
GOTERM_BP_DIRECT	kinetochore assembly	4	567	13	19512	5.59E-03	1.80E-01	10.59	1.00E+00	1.76E-01	4.09E-04
GOTERM_BP_DIRECT	peptidyl-serine phosphorylation	7	567	48	19512	2.53E-03	9.41E-02	5.02	9.99E-01	9.20E-02	4.41E-04
GOTERM_BP_DIRECT	blastocyst formation	5	567	23	19512	4.00E-03	1.37E-01	7.48	1.00E+00	1.34E-01	4.44E-04
GOTERM_BP_DIRECT	thymidine biosynthetic process	3	567	6	19512	1.17E-02	2.99E-01	17.21	1.00E+00	2.93E-01	4.57E-04
GOTERM_BP_DIRECT	Golgi disassembly	3	567	6	19512	1.17E-02	2.99E-01	17.21	1.00E+00	2.93E-01	4.57E-04
GOTERM_BP_DIRECT	nucleotide biosynthetic process	4	567	14	19512	6.96E-03	2.08E-01	9.83	1.00E+00	2.04E-01	5.60E-04
GOTERM_BP_DIRECT	somatic hypermutation of immunoglobulin genes	4	567	14	19512	6.96E-03	2.08E-01	9.83	1.00E+00	2.04E-01	5.60E-04
GOTERM_BP_DIRECT	regulation of microtubule-based process	4	567	14	19512	6.96E-03	2.08E-01	9.83	1.00E+00	2.04E-01	5.60E-04
GOTERM_BP_DIRECT	microtubule depolymerization	4	567	15	19512	8.52E-03	2.41E-01	9.18	1.00E+00	2.36E-01	7.46E-04
GOTERM_BP_DIRECT	negative regulation of cytokinesis	3	567	7	19512	1.60E-02	3.79E-01	14.75	1.00E+00	3.71E-01	7.83E-04
GOTERM_BP_DIRECT	DNA replication, synthesis of primer	3	567	7	19512	1.60E-02	3.79E-01	14.75	1.00E+00	3.71E-01	7.83E-04
GOTERM_BP_DIRECT	regulation of meiotic nuclear division	3	567	7	19512	1.60E-02	3.79E-01	14.75	1.00E+00	3.71E-01	7.83E-04
GOTERM_BP_DIRECT	mitotic recombination	3	567	7	19512	1.60E-02	3.79E-01	14.75	1.00E+00	3.71E-01	7.83E-04
GOTERM_BP_DIRECT	positive regulation of transcription by RNA polymerase I	5	567	26	19512	6.31E-03	1.98E-01	6.62	1.00E+00	1.93E-01	8.07E-04
GOTERM_BP_DIRECT	urate biosynthetic process	2	567	2	19512	5.72E-02	8.97E-01	34.41	1.00E+00	8.77E-01	8.43E-04
GOTERM_BP_DIRECT	broken chromosome clustering	2	567	2	19512	5.72E-02	8.97E-01	34.41	1.00E+00	8.77E-01	8.43E-04

Category	Term	Count	List Total	Pop Hits	Pop Total	P-Value	Benjamini	Fold Enrichment	Bonferroni	FDR	Fisher Exact
GOTERM_BP_DIRECT	dichotomous subdivision of terminal units involved in mammary gland duct morphogenesis	2	567	2	19512	5.72E-02	8.97E-01	34.41	1.00E+00	8.77E-01	8.43E-04
GOTERM_BP_DIRECT	cellular response to bleomycin	2	567	2	19512	5.72E-02	8.97E-01	34.41	1.00E+00	8.77E-01	8.43E-04
GOTERM_BP_DIRECT	repair of mitotic kinetochore microtubule attachment defect	2	567	2	19512	5.72E-02	8.97E-01	34.41	1.00E+00	8.77E-01	8.43E-04
GOTERM_BP_DIRECT	mitotic spindle elongation	2	567	2	19512	5.72E-02	8.97E-01	34.41	1.00E+00	8.77E-01	8.43E-04
GOTERM_BP_DIRECT	base-excision repair	6	567	39	19512	5.14E-03	1.67E-01	5.29	1.00E+00	1.64E-01	8.46E-04
GOTERM_BP_DIRECT	interstrand cross-link repair	6	567	39	19512	5.14E-03	1.67E-01	5.29	1.00E+00	1.64E-01	8.46E-04
GOTERM_BP_DIRECT	regulation of mitotic spindle assembly	5	567	27	19512	7.24E-03	2.14E-01	6.37	1.00E+00	2.09E-01	9.67E-04
GOTERM_BP_DIRECT	negative regulation of gene expression via chromosomal CpG island methylation	4	567	16	19512	1.03E-02	2.84E-01	8.6	1.00E+00	2.78E-01	9.72E-04
GOTERM_BP_DIRECT	roof of mouth development	8	567	71	19512	4.46E-03	1.51E-01	3.88	1.00E+00	1.47E-01	1.03E-03
GOTERM_BP_DIRECT	positive regulation of cytokinesis	6	567	41	19512	6.39E-03	1.98E-01	5.04	1.00E+00	1.94E-01	1.11E-03
GOTERM_BP_DIRECT	chromosome organization involved in meiotic cell cycle	3	567	8	19512	2.09E-02	4.51E-01	12.9	1.00E+00	4.41E-01	1.23E-03
GOTERM_BP_DIRECT	positive regulation of spindle assembly	3	567	8	19512	2.09E-02	4.51E-01	12.9	1.00E+00	4.41E-01	1.23E-03
GOTERM_BP_DIRECT	regulation of phosphorylation	3	567	8	19512	2.09E-02	4.51E-01	12.9	1.00E+00	4.41E-01	1.23E-03
GOTERM_BP_DIRECT	mitotic intra-S DNA damage checkpoint signaling	4	567	17	19512	1.22E-02	3.05E-01	8.1	1.00E+00	2.98E-01	1.24E-03
GOTERM_BP_DIRECT	positive regulation of cell population proliferation	29	567	542	19512	2.46E-03	9.30E-02	1.84	9.98E-01	9.09E-02	1.26E-03
GOTERM_BP_DIRECT	rRNA processing	13	567	169	19512	3.90E-03	1.35E-01	2.65	1.00E+00	1.32E-01	1.36E-03
GOTERM_BP_DIRECT	regulation of mitotic cell cycle	8	567	75	19512	6.03E-03	1.91E-01	3.67	1.00E+00	1.87E-01	1.48E-03
GOTERM_BP_DIRECT	chromatin remodeling	20	567	328	19512	3.50E-03	1.23E-01	2.1	1.00E+00	1.20E-01	1.56E-03
GOTERM_BP_DIRECT	positive regulation of transcription by RNA polymerase III	4	567	18	19512	1.43E-02	3.52E-01	7.65	1.00E+00	3.44E-01	1.56E-03
GOTERM_BP_DIRECT	regulation of DNA replication	6	567	45	19512	9.47E-03	2.65E-01	4.59	1.00E+00	2.59E-01	1.82E-03

Category	Term	Count	List Total	Pop Hits	Pop Total	P-Value	Benjamini	Fold Enrichment	Bonferroni	FDR	Fisher Exact
GOTERM_BP_DIRECT	oocyte maturation	5	567	31	19512	1.18E-02	2.99E-01	5.55	1.00E+00	2.93E-01	1.85E-03
GOTERM_BP_DIRECT	DNA synthesis involved in DNA repair	5	567	31	19512	1.18E-02	2.99E-01	5.55	1.00E+00	2.93E-01	1.85E-03
GOTERM_BP_DIRECT	response to X-ray	4	567	19	19512	1.66E-02	3.80E-01	7.24	1.00E+00	3.72E-01	1.93E-03
GOTERM_BP_DIRECT	centrosome duplication	4	567	19	19512	1.66E-02	3.80E-01	7.24	1.00E+00	3.72E-01	1.93E-03
GOTERM_BP_DIRECT	attachment of mitotic spindle microtubules to kinetochore	4	567	19	19512	1.66E-02	3.80E-01	7.24	1.00E+00	3.72E-01	1.93E-03
GOTERM_BP_DIRECT	positive regulation of double-strand break repair	6	567	47	19512	1.13E-02	2.99E-01	4.39	1.00E+00	2.93E-01	2.29E-03
GOTERM_BP_DIRECT	DNA damage checkpoint signaling	6	567	47	19512	1.13E-02	2.99E-01	4.39	1.00E+00	2.93E-01	2.29E-03
GOTERM_BP_DIRECT	skeletal system morphogenesis	6	567	47	19512	1.13E-02	2.99E-01	4.39	1.00E+00	2.93E-01	2.29E-03
GOTERM_BP_DIRECT	response to radiation	4	567	20	19512	1.92E-02	4.24E-01	6.88	1.00E+00	4.15E-01	2.36E-03
GOTERM_BP_DIRECT	isotype switching	4	567	20	19512	1.92E-02	4.24E-01	6.88	1.00E+00	4.15E-01	2.36E-03
GOTERM_BP_DIRECT	nuclear DNA replication	2	567	3	19512	8.45E-02	1.00E+00	22.94	1.00E+00	9.78E-01	2.48E-03
GOTERM_BP_DIRECT	spindle elongation	2	567	3	19512	8.45E-02	1.00E+00	22.94	1.00E+00	9.78E-01	2.48E-03
GOTERM_BP_DIRECT	chemokine (C-X-C motif) ligand 12 signaling pathway	2	567	3	19512	8.45E-02	1.00E+00	22.94	1.00E+00	9.78E-01	2.48E-03
GOTERM_BP_DIRECT	negative regulation of mitotic metaphase/anaphase transition	2	567	3	19512	8.45E-02	1.00E+00	22.94	1.00E+00	9.78E-01	2.48E-03
GOTERM_BP_DIRECT	pericentric heterochromatin organization	2	567	3	19512	8.45E-02	1.00E+00	22.94	1.00E+00	9.78E-01	2.48E-03
GOTERM_BP_DIRECT	positive regulation of mitotic sister chromatid segregation	2	567	3	19512	8.45E-02	1.00E+00	22.94	1.00E+00	9.78E-01	2.48E-03
GOTERM_BP_DIRECT	negative regulation of supramolecular fiber organization	2	567	3	19512	8.45E-02	1.00E+00	22.94	1.00E+00	9.78E-01	2.48E-03
GOTERM_BP_DIRECT	post-translational protein acetylation	2	567	3	19512	8.45E-02	1.00E+00	22.94	1.00E+00	9.78E-01	2.48E-03
GOTERM_BP_DIRECT	mitotic cell cycle phase transition	3	567	10	19512	3.24E-02	6.44E-01	10.32	1.00E+00	6.30E-01	2.51E-03
GOTERM_BP_DIRECT	platelet-derived growth factor receptor signaling pathway	5	567	34	19512	1.63E-02	3.80E-01	5.06	1.00E+00	3.72E-01	2.82E-03
GOTERM_BP_DIRECT	regulation of mitotic spindle organization	4	567	21	19512	2.19E-02	4.60E-01	6.55	1.00E+00	4.49E-01	2.85E-03

Category	Term	Count	List Total	Pop Hits	Pop Total	P-Value	Benjamini	Fold Enrichment	Bonferroni	FDR	Fisher Exact
GOTERM_BP_DIRECT	positive regulation of protein catabolic process	8	567	84	19512	1.10E-02	2.99E-01	3.28	1.00E+00	2.93E-01	3.04E-03
GOTERM_BP_DIRECT	telomere maintenance	7	567	67	19512	1.30E-02	3.22E-01	3.6	1.00E+00	3.15E-01	3.25E-03
GOTERM_BP_DIRECT	regulation of cell cycle phase transition	3	567	11	19512	3.88E-02	7.12E-01	9.39	1.00E+00	6.96E-01	3.38E-03
GOTERM_BP_DIRECT	phosphate ion transport	3	567	11	19512	3.88E-02	7.12E-01	9.39	1.00E+00	6.96E-01	3.38E-03
GOTERM_BP_DIRECT	mitotic nuclear membrane disassembly	3	567	11	19512	3.88E-02	7.12E-01	9.39	1.00E+00	6.96E-01	3.38E-03
GOTERM_BP_DIRECT	cellular response to hydroxyurea	3	567	11	19512	3.88E-02	7.12E-01	9.39	1.00E+00	6.96E-01	3.38E-03
GOTERM_BP_DIRECT	regulation of cyclin-dependent protein serine/threonine kinase activity	4	567	22	19512	2.48E-02	5.08E-01	6.26	1.00E+00	4.96E-01	3.40E-03
GOTERM_BP_DIRECT	regulation of cell cycle process	4	567	23	19512	2.79E-02	5.62E-01	5.98	1.00E+00	5.50E-01	4.03E-03
GOTERM_BP_DIRECT	positive regulation of cell migration involved in sprouting angiogenesis	5	567	37	19512	2.17E-02	4.60E-01	4.65	1.00E+00	4.49E-01	4.11E-03
GOTERM_BP_DIRECT	positive regulation of G1/S transition of mitotic cell cycle	6	567	53	19512	1.84E-02	4.18E-01	3.9	1.00E+00	4.08E-01	4.23E-03
GOTERM_BP_DIRECT	regulation of chromosome segregation	3	567	12	19512	4.57E-02	8.21E-01	8.6	1.00E+00	8.03E-01	4.41E-03
GOTERM_BP_DIRECT	DNA damage tolerance	3	567	12	19512	4.57E-02	8.21E-01	8.6	1.00E+00	8.03E-01	4.41E-03
GOTERM_BP_DIRECT	positive regulation of fibroblast proliferation	6	567	55	19512	2.13E-02	4.55E-01	3.75	1.00E+00	4.45E-01	5.09E-03
GOTERM_BP_DIRECT	decidualization	4	567	25	19512	3.48E-02	6.71E-01	5.51	1.00E+00	6.56E-01	5.49E-03
GOTERM_BP_DIRECT	cellular hyperosmotic response	3	567	13	19512	5.30E-02	8.78E-01	7.94	1.00E+00	8.58E-01	5.62E-03
GOTERM_BP_DIRECT	tetrahydrofolate metabolic process	3	567	13	19512	5.30E-02	8.78E-01	7.94	1.00E+00	8.58E-01	5.62E-03
GOTERM_BP_DIRECT	phosphate ion transmembrane transport	3	567	13	19512	5.30E-02	8.78E-01	7.94	1.00E+00	8.58E-01	5.62E-03
GOTERM_BP_DIRECT	random inactivation of X chromosome	3	567	13	19512	5.30E-02	8.78E-01	7.94	1.00E+00	8.58E-01	5.62E-03
GOTERM_BP_DIRECT	nitric oxide mediated signal transduction	3	567	13	19512	5.30E-02	8.78E-01	7.94	1.00E+00	8.58E-01	5.62E-03
GOTERM_BP_DIRECT	regulation of cell communication	3	567	13	19512	5.30E-02	8.78E-01	7.94	1.00E+00	8.58E-01	5.62E-03

Category	Term	Count	List Total	Pop Hits	Pop Total	P-Value	Benjamini	Fold Enrichment	Bonferroni	FDR	Fisher Exact
GOTERM_BP_DIRECT	regulation of cytokinesis	5	567	40	19512	2.81E-02	5.62E-01	4.3	1.00E+00	5.50E-01	5.78E-03
GOTERM_BP_DIRECT	inhibitory synapse assembly	4	567	26	19512	3.85E-02	7.12E-01	5.29	1.00E+00	6.96E-01	6.34E-03
GOTERM_BP_DIRECT	intracellular signal transduction	25	567	501	19512	1.17E-02	2.99E-01	1.72	1.00E+00	2.93E-01	6.43E-03
GOTERM_BP_DIRECT	trophoblast giant cell differentiation	3	567	14	19512	6.07E-02	9.30E-01	7.37	1.00E+00	9.09E-01	6.99E-03
GOTERM_BP_DIRECT	single strand break repair	3	567	14	19512	6.07E-02	9.30E-01	7.37	1.00E+00	9.09E-01	6.99E-03
GOTERM_BP_DIRECT	activation of protein kinase activity	3	567	14	19512	6.07E-02	9.30E-01	7.37	1.00E+00	9.09E-01	6.99E-03
GOTERM_BP_DIRECT	spindle assembly	5	567	42	19512	3.29E-02	6.49E-01	4.1	1.00E+00	6.34E-01	7.13E-03
GOTERM_BP_DIRECT	nucleosome assembly	11	567	161	19512	1.92E-02	4.24E-01	2.35	1.00E+00	4.15E-01	7.51E-03
GOTERM_BP_DIRECT	cellular response to ionizing radiation	5	567	43	19512	3.55E-02	6.74E-01	4	1.00E+00	6.59E-01	7.88E-03
GOTERM_BP_DIRECT	DNA metabolic process	5	567	43	19512	3.55E-02	6.74E-01	4	1.00E+00	6.59E-01	7.88E-03
GOTERM_BP_DIRECT	cell volume homeostasis	4	567	28	19512	4.65E-02	8.29E-01	4.92	1.00E+00	8.11E-01	8.30E-03
GOTERM_BP_DIRECT	microtubule anchoring	3	567	15	19512	6.87E-02	1.00E+00	6.88	1.00E+00	9.78E-01	8.56E-03
GOTERM_BP_DIRECT	inner cell mass cell proliferation	3	567	15	19512	6.87E-02	1.00E+00	6.88	1.00E+00	9.78E-01	8.56E-03
GOTERM_BP_DIRECT	negative regulation of G2/M transition of mitotic cell cycle	3	567	15	19512	6.87E-02	1.00E+00	6.88	1.00E+00	9.78E-01	8.56E-03
GOTERM_BP_DIRECT	nucleobase-containing compound metabolic process	6	567	62	19512	3.37E-02	6.60E-01	3.33	1.00E+00	6.46E-01	9.10E-03
GOTERM_BP_DIRECT	male meiosis I	4	567	29	19512	5.08E-02	8.78E-01	4.75	1.00E+00	8.58E-01	9.41E-03
GOTERM_BP_DIRECT	blastocyst development	4	567	29	19512	5.08E-02	8.78E-01	4.75	1.00E+00	8.58E-01	9.41E-03
GOTERM_BP_DIRECT	negative regulation of intrinsic apoptotic signaling pathway in response to DNA damage by p53 class mediator	3	567	16	19512	7.71E-02	1.00E+00	6.45	1.00E+00	9.78E-01	1.03E-02
GOTERM_BP_DIRECT	regulation of signaling	3	567	16	19512	7.71E-02	1.00E+00	6.45	1.00E+00	9.78E-01	1.03E-02
GOTERM_BP_DIRECT	positive regulation of blood coagulation	3	567	16	19512	7.71E-02	1.00E+00	6.45	1.00E+00	9.78E-01	1.03E-02
GOTERM_BP_DIRECT	hyaluronan catabolic process	3	567	16	19512	7.71E-02	1.00E+00	6.45	1.00E+00	9.78E-01	1.03E-02
GOTERM_BP_DIRECT	methylation	13	567	215	19512	2.34E-02	4.88E-01	2.08	1.00E+00	4.77E-01	1.04E-02
GOTERM_BP_DIRECT	response to ionizing radiation	5	567	46	19512	4.38E-02	7.98E-01	3.74	1.00E+00	7.81E-01	1.05E-02
GOTERM_BP_DIRECT	cardiac muscle cell differentiation	4	567	30	19512	5.52E-02	8.97E-01	4.59	1.00E+00	8.77E-01	1.06E-02

Category	Term	Count	List Total	Pop Hits	Pop Total	P-Value	Benjamini	Fold Enrichment	Bonferroni	FDR	Fisher Exact
GOTERM_BP_DIRECT	negative regulation of extrinsic apoptotic signaling pathway via death domain receptors	4	567	30	19512	5.52E-02	8.97E-01	4.59	1.00E+00	8.77E-01	1.06E-02
GOTERM_BP_DIRECT	cell population proliferation	15	567	267	19512	2.45E-02	5.06E-01	1.93	1.00E+00	4.95E-01	1.17E-02
GOTERM_BP_DIRECT	mitotic G1 DNA damage checkpoint signaling	3	567	17	19512	8.58E-02	1.00E+00	6.07	1.00E+00	9.78E-01	1.22E-02
GOTERM_BP_DIRECT	cellular response to nitric oxide	3	567	17	19512	8.58E-02	1.00E+00	6.07	1.00E+00	9.78E-01	1.22E-02
GOTERM_BP_DIRECT	regulation of dendrite development	3	567	17	19512	8.58E-02	1.00E+00	6.07	1.00E+00	9.78E-01	1.22E-02
GOTERM_BP_DIRECT	dendritic spine development	3	567	17	19512	8.58E-02	1.00E+00	6.07	1.00E+00	9.78E-01	1.22E-02
GOTERM_BP_DIRECT	response to virus	9	567	129	19512	3.44E-02	6.69E-01	2.4	1.00E+00	6.54E-01	1.31E-02
GOTERM_BP_DIRECT	positive regulation of cardiac muscle cell proliferation	4	567	32	19512	6.47E-02	9.76E-01	4.3	1.00E+00	9.54E-01	1.33E-02
GOTERM_BP_DIRECT	regulation of neurogenesis	5	567	49	19512	5.32E-02	8.78E-01	3.51	1.00E+00	8.58E-01	1.36E-02
GOTERM_BP_DIRECT	mRNA processing	21	567	427	19512	2.50E-02	5.08E-01	1.69	1.00E+00	4.96E-01	1.38E-02
GOTERM_BP_DIRECT	face morphogenesis	4	567	33	19512	6.97E-02	1.00E+00	4.17	1.00E+00	9.78E-01	1.48E-02
GOTERM_BP_DIRECT	regulation of circadian rhythm	6	567	69	19512	4.98E-02	8.77E-01	2.99	1.00E+00	8.57E-01	1.50E-02
GOTERM_BP_DIRECT	cerebral cortex development	7	567	91	19512	4.90E-02	8.67E-01	2.65	1.00E+00	8.48E-01	1.67E-02
GOTERM_BP_DIRECT	centrosome cycle	5	567	52	19512	6.37E-02	9.69E-01	3.31	1.00E+00	9.48E-01	1.72E-02
GOTERM_BP_DIRECT	cochlea development	4	567	35	19512	8.02E-02	1.00E+00	3.93	1.00E+00	9.78E-01	1.81E-02
GOTERM_BP_DIRECT	cellular response to hypoxia	9	567	140	19512	5.15E-02	8.78E-01	2.21	1.00E+00	8.58E-01	2.12E-02
GOTERM_BP_DIRECT	establishment of mitotic spindle orientation	4	567	37	19512	9.14E-02	1.00E+00	3.72	1.00E+00	9.78E-01	2.18E-02
GOTERM_BP_DIRECT	intrinsic apoptotic signaling pathway in response to DNA damage	5	567	56	19512	7.91E-02	1.00E+00	3.07	1.00E+00	9.78E-01	2.30E-02
GOTERM_BP_DIRECT	positive regulation of erythrocyte differentiation	4	567	38	19512	9.72E-02	1.00E+00	3.62	1.00E+00	9.78E-01	2.39E-02
GOTERM_BP_DIRECT	post-translational protein modification	4	567	38	19512	9.72E-02	1.00E+00	3.62	1.00E+00	9.78E-01	2.39E-02
GOTERM_BP_DIRECT	positive regulation of multicellular organismal process	4	567	38	19512	9.72E-02	1.00E+00	3.62	1.00E+00	9.78E-01	2.39E-02
GOTERM_BP_DIRECT	response to axon injury	4	567	38	19512	9.72E-02	1.00E+00	3.62	1.00E+00	9.78E-01	2.39E-02

Category	Term	Count	List Total	Pop Hits	Pop Total	P-Value	Benjamini	Fold Enrichment	Bonferroni	FDR	Fisher Exact
GOTERM_BP_DIRECT	ribosome biogenesis	8	567	122	19512	6.49E-02	9.76E-01	2.26	1.00E+00	9.54E-01	2.61E-02
GOTERM_BP_DIRECT	negative regulation of G1/S transition of mitotic cell cycle	5	567	58	19512	8.74E-02	1.00E+00	2.97	1.00E+00	9.78E-01	2.64E-02
GOTERM_BP_DIRECT	negative regulation of cell cycle	5	567	59	19512	9.17E-02	1.00E+00	2.92	1.00E+00	9.78E-01	2.82E-02
GOTERM_BP_DIRECT	RNA splicing	16	567	329	19512	5.77E-02	8.99E-01	1.67	1.00E+00	8.79E-01	3.17E-02
GOTERM_BP_DIRECT	wound healing	6	567	82	19512	8.98E-02	1.00E+00	2.52	1.00E+00	9.78E-01	3.21E-02
GOTERM_BP_DIRECT	rhythmic process	9	567	151	19512	7.33E-02	1.00E+00	2.05	1.00E+00	9.78E-01	3.25E-02
GOTERM_BP_DIRECT	response to hypoxia	10	567	181	19512	8.17E-02	1.00E+00	1.9	1.00E+00	9.78E-01	3.91E-02
GOTERM_BP_DIRECT	response to xenobiotic stimulus	13	567	260	19512	7.81E-02	1.00E+00	1.72	1.00E+00	9.78E-01	4.11E-02
GOTERM_BP_DIRECT	negative regulation of apoptotic process	23	567	543	19512	7.36E-02	1.00E+00	1.46	1.00E+00	9.78E-01	4.62E-02
GOTERM_BP_DIRECT	angiogenesis	14	567	292	19512	8.63E-02	1.00E+00	1.65	1.00E+00	9.78E-01	4.67E-02
GOTERM_BP_DIRECT	microtubule cytoskeleton organization	9	567	162	19512	9.99E-02	1.00E+00	1.91	1.00E+00	9.78E-01	4.73E-02

Table S4 GO terms associated with upregulated DEGs without H3K4me3-gain.

Category	Term	Count	List Total	Pop Hits	Pop Total	P-Value	Benjamini	Fold Enrichment	Bonferroni	FDR	Fisher Exact
GOTERM_BP_DIRECT	extracellular matrix organization	23	712	147	19512	1.84E-08	3.18E-05	4.29	6.35E-05	3.16E-05	3.74E-09
GOTERM_BP_DIRECT	lipid metabolic process	66	712	847	19512	1.15E-08	3.18E-05	2.14	3.99E-05	3.16E-05	5.13E-09
GOTERM_BP_DIRECT	steroid biosynthetic process	15	712	65	19512	6.98E-08	8.04E-05	6.32	2.41E-04	7.99E-05	8.89E-09
GOTERM_BP_DIRECT	cholesterol biosynthetic process	12	712	41	19512	1.62E-07	1.40E-04	8.02	5.60E-04	1.39E-04	1.52E-08
GOTERM_BP_DIRECT	cholesterol metabolic process	18	712	109	19512	4.15E-07	2.39E-04	4.53	1.43E-03	2.37E-04	7.92E-08
GOTERM_BP_DIRECT	sterol biosynthetic process	10	712	32	19512	1.42E-06	7.02E-04	8.56	4.90E-03	6.97E-04	1.22E-07
GOTERM_BP_DIRECT	cell adhesion	53	712	677	19512	3.45E-07	2.39E-04	2.15	1.19E-03	2.37E-04	1.53E-07
GOTERM_BP_DIRECT	opsonization	7	712	18	19512	2.92E-05	1.01E-02	10.66	9.61E-02	1.00E-02	1.87E-06
GOTERM_BP_DIRECT	activation of membrane attack complex	6	712	13	19512	6.39E-05	1.70E-02	12.65	1.98E-01	1.69E-02	3.19E-06
GOTERM_BP_DIRECT	lipid transport	21	712	193	19512	2.70E-05	1.01E-02	2.98	8.90E-02	1.00E-02	8.24E-06
GOTERM_BP_DIRECT	isoprenoid biosynthetic process	6	712	15	19512	1.40E-04	3.12E-02	10.96	3.84E-01	3.10E-02	8.73E-06
GOTERM_BP_DIRECT	cell-matrix adhesion	15	712	108	19512	3.77E-05	1.18E-02	3.81	1.22E-01	1.18E-02	8.77E-06
GOTERM_BP_DIRECT	cell-cell adhesion	23	712	225	19512	2.70E-05	1.01E-02	2.8	8.91E-02	1.00E-02	8.83E-06
GOTERM_BP_DIRECT	steroid metabolic process	17	712	138	19512	4.37E-05	1.26E-02	3.38	1.40E-01	1.25E-02	1.16E-05
GOTERM_BP_DIRECT	Wnt signaling pathway, planar cell polarity pathway	8	712	34	19512	1.89E-04	3.83E-02	6.45	4.79E-01	3.81E-02	2.36E-05
GOTERM_BP_DIRECT	positive regulation of neuron projection development	15	712	122	19512	1.44E-04	3.12E-02	3.37	3.93E-01	3.10E-02	3.85E-05
GOTERM_BP_DIRECT	bone mineralization	10	712	58	19512	2.32E-04	4.21E-02	4.72	5.51E-01	4.19E-02	4.20E-05
GOTERM_BP_DIRECT	carbohydrate metabolic process	19	712	184	19512	1.41E-04	3.12E-02	2.83	3.86E-01	3.10E-02	4.55E-05
GOTERM_BP_DIRECT	phospholipid transport	9	712	48	19512	3.10E-04	5.10E-02	5.14	6.58E-01	5.07E-02	5.10E-05
GOTERM_BP_DIRECT	complement activation	8	712	38	19512	3.90E-04	5.86E-02	5.77	7.40E-01	5.82E-02	5.60E-05
GOTERM_BP_DIRECT	response to ethanol	14	712	112	19512	2.20E-04	4.21E-02	3.43	5.33E-01	4.19E-02	5.76E-05
GOTERM_BP_DIRECT	complement activation, lectin pathway	5	712	13	19512	9.61E-04	1.19E-01	10.54	9.64E-01	1.18E-01	6.43E-05
GOTERM_BP_DIRECT	negative regulation of cell adhesion	9	712	51	19512	4.75E-04	6.57E-02	4.84	8.07E-01	6.53E-02	8.39E-05
GOTERM_BP_DIRECT	peptidyl-tyrosine phosphorylation	8	712	41	19512	6.32E-04	8.17E-02	5.35	8.87E-01	8.12E-02	9.93E-05
GOTERM_BP_DIRECT	axon guidance	19	712	198	19512	3.50E-04	5.50E-02	2.63	7.02E-01	5.47E-02	1.22E-04
GOTERM_BP_DIRECT	proteolysis	44	712	671	19512	2.54E-04	4.38E-02	1.8	5.84E-01	4.35E-02	1.34E-04

GOTERM_BP_DIRECT	insulin receptor signaling pathway	11	712	80	19512	6.39E-04	8.17E-02	3.77	8.90E-01	8.12E-02	1.50E-04
GOTERM_BP_DIRECT	bone morphogenesis	7	712	34	19512	1.29E-03	1.39E-01	5.64	9.88E-01	1.38E-01	1.90E-04
GOTERM_BP_DIRECT	semaphorin-plexin signaling pathway	8	712	45	19512	1.12E-03	1.30E-01	4.87	9.80E-01	1.29E-01	1.97E-04
GOTERM_BP_DIRECT	extracellular matrix disassembly	8	712	46	19512	1.29E-03	1.39E-01	4.77	9.88E-01	1.38E-01	2.31E-04
GOTERM_BP_DIRECT	nervous system development	42	712	649	19512	4.65E-04	6.57E-02	1.77	7.99E-01	6.53E-02	2.52E-04
GOTERM_BP_DIRECT	peptide catabolic process	5	712	17	19512	2.85E-03	2.66E-01	8.06	1.00E+00	2.64E-01	2.74E-04
GOTERM_BP_DIRECT	muscle organ development	13	712	115	19512	9.99E-04	1.19E-01	3.1	9.68E-01	1.18E-01	2.91E-04
GOTERM_BP_DIRECT	bile acid biosynthetic process	6	712	27	19512	2.63E-03	2.52E-01	6.09	1.00E+00	2.50E-01	3.55E-04
GOTERM_BP_DIRECT	muscular septum morphogenesis	3	712	5	19512	1.23E-02	6.87E-01	16.44	1.00E+00	6.82E-01	4.58E-04
GOTERM_BP_DIRECT	neuronal action potential propagation	4	712	11	19512	6.39E-03	4.77E-01	9.97	1.00E+00	4.74E-01	4.73E-04
GOTERM_BP_DIRECT	response to lipid	5	712	19	19512	4.38E-03	3.52E-01	7.21	1.00E+00	3.49E-01	4.84E-04
GOTERM_BP_DIRECT	positive regulation of axonogenesis	6	712	30	19512	4.24E-03	3.49E-01	5.48	1.00E+00	3.46E-01	6.48E-04
GOTERM_BP_DIRECT	inflammatory response to wounding	4	712	12	19512	8.29E-03	5.42E-01	9.13	1.00E+00	5.38E-01	6.88E-04
GOTERM_BP_DIRECT	response to glucocorticoid	8	712	54	19512	3.31E-03	2.97E-01	4.06	1.00E+00	2.95E-01	7.12E-04
GOTERM_BP_DIRECT	bile acid secretion	3	712	6	19512	1.80E-02	8.56E-01	13.7	1.00E+00	8.50E-01	8.91E-04
GOTERM_BP_DIRECT	positive regulation of inhibitory postsynaptic potential	3	712	6	19512	1.80E-02	8.56E-01	13.7	1.00E+00	8.50E-01	8.91E-04
GOTERM_BP_DIRECT	muscle cell fate commitment	3	712	6	19512	1.80E-02	8.56E-01	13.7	1.00E+00	8.50E-01	8.91E-04
GOTERM_BP_DIRECT	cell migration	23	712	306	19512	1.99E-03	2.02E-01	2.06	9.99E-01	2.01E-01	9.00E-04
GOTERM_BP_DIRECT	sterol metabolic process	5	712	22	19512	7.58E-03	5.24E-01	6.23	1.00E+00	5.20E-01	1.00E-03
GOTERM_BP_DIRECT	transmembrane transport	36	712	569	19512	1.83E-03	1.92E-01	1.73	9.98E-01	1.90E-01	1.01E-03
GOTERM_BP_DIRECT	transport across blood-brain barrier	10	712	85	19512	3.75E-03	3.17E-01	3.22	1.00E+00	3.14E-01	1.04E-03
GOTERM_BP_DIRECT	lipid biosynthetic process	6	712	33	19512	6.45E-03	4.77E-01	4.98	1.00E+00	4.74E-01	1.10E-03
GOTERM_BP_DIRECT	angiogenesis	22	712	292	19512	2.48E-03	2.45E-01	2.06	1.00E+00	2.43E-01	1.12E-03
GOTERM_BP_DIRECT	complement activation, classical pathway	7	712	45	19512	5.56E-03	4.37E-01	4.26	1.00E+00	4.34E-01	1.13E-03
GOTERM_BP_DIRECT	cytoskeleton organization	15	712	168	19512	3.44E-03	2.97E-01	2.45	1.00E+00	2.95E-01	1.29E-03
GOTERM_BP_DIRECT	gamma-aminobutyric acid catabolic process	2	712	2	19512	7.16E-02	1.00E+00	27.4	1.00E+00	9.94E-01	1.33E-03
GOTERM_BP_DIRECT	fasciculation of motor neuron axon	2	712	2	19512	7.16E-02	1.00E+00	27.4	1.00E+00	9.94E-01	1.33E-03
GOTERM_BP_DIRECT	focal adhesion disassembly	2	712	2	19512	7.16E-02	1.00E+00	27.4	1.00E+00	9.94E-01	1.33E-03

GOTERM_BP_DIRECT	regulation of phospholipid biosynthetic process	2	712	2	19512	7.16E-02	1.00E+00	27.4	1.00E+00	9.94E-01	1.33E-03
GOTERM_BP_DIRECT	negative regulation of gamma-aminobutyric acid secretion	2	712	2	19512	7.16E-02	1.00E+00	27.4	1.00E+00	9.94E-01	1.33E-03
GOTERM_BP_DIRECT	disaccharide metabolic process	2	712	2	19512	7.16E-02	1.00E+00	27.4	1.00E+00	9.94E-01	1.33E-03
GOTERM_BP_DIRECT	Leydig cell proliferation	2	712	2	19512	7.16E-02	1.00E+00	27.4	1.00E+00	9.94E-01	1.33E-03
GOTERM_BP_DIRECT	regulation of hepatocyte proliferation	2	712	2	19512	7.16E-02	1.00E+00	27.4	1.00E+00	9.94E-01	1.33E-03
GOTERM_BP_DIRECT	neural crest cell migration	7	712	47	19512	6.90E-03	4.97E-01	4.08	1.00E+00	4.93E-01	1.48E-03
GOTERM_BP_DIRECT	zymogen activation	6	712	35	19512	8.31E-03	5.42E-01	4.7	1.00E+00	5.38E-01	1.52E-03
GOTERM_BP_DIRECT	cardiac muscle cell action potential	3	712	7	19512	2.47E-02	1.00E+00	11.74	1.00E+00	9.94E-01	1.52E-03
GOTERM_BP_DIRECT	recognition of apoptotic cell	3	712	7	19512	2.47E-02	1.00E+00	11.74	1.00E+00	9.94E-01	1.52E-03
GOTERM_BP_DIRECT	positive regulation of ceramide biosynthetic process	3	712	7	19512	2.47E-02	1.00E+00	11.74	1.00E+00	9.94E-01	1.52E-03
GOTERM_BP_DIRECT	positive regulation of cell migration	22	712	300	19512	3.41E-03	2.97E-01	2.01	1.00E+00	2.95E-01	1.58E-03
GOTERM_BP_DIRECT	short-term memory	4	712	15	19512	1.58E-02	8.31E-01	7.31	1.00E+00	8.26E-01	1.74E-03
GOTERM_BP_DIRECT	myotube differentiation	4	712	16	19512	1.89E-02	8.64E-01	6.85	1.00E+00	8.58E-01	2.25E-03
GOTERM_BP_DIRECT	phosphorylation	4	712	16	19512	1.89E-02	8.64E-01	6.85	1.00E+00	8.58E-01	2.25E-03
GOTERM_BP_DIRECT	response to pH	3	712	8	19512	3.21E-02	1.00E+00	10.28	1.00E+00	9.94E-01	2.36E-03
GOTERM_BP_DIRECT	response to folic acid	3	712	8	19512	3.21E-02	1.00E+00	10.28	1.00E+00	9.94E-01	2.36E-03
GOTERM_BP_DIRECT	cellular response to interleukin-17	3	712	8	19512	3.21E-02	1.00E+00	10.28	1.00E+00	9.94E-01	2.36E-03
GOTERM_BP_DIRECT	Peyer's patch development	3	712	8	19512	3.21E-02	1.00E+00	10.28	1.00E+00	9.94E-01	2.36E-03
GOTERM_BP_DIRECT	lysosome organization	8	712	65	19512	9.23E-03	5.80E-01	3.37	1.00E+00	5.76E-01	2.43E-03
GOTERM_BP_DIRECT	positive regulation of cholesterol efflux	5	712	27	19512	1.57E-02	8.31E-01	5.07	1.00E+00	8.26E-01	2.64E-03
GOTERM_BP_DIRECT	response to nutrient	8	712	66	19512	1.00E-02	6.07E-01	3.32	1.00E+00	6.03E-01	2.68E-03
GOTERM_BP_DIRECT	inner ear morphogenesis	8	712	66	19512	1.00E-02	6.07E-01	3.32	1.00E+00	6.03E-01	2.68E-03
GOTERM_BP_DIRECT	ephrin receptor signaling pathway	7	712	52	19512	1.12E-02	6.58E-01	3.69	1.00E+00	6.54E-01	2.68E-03
GOTERM_BP_DIRECT	lipid catabolic process	12	712	130	19512	8.02E-03	5.42E-01	2.53	1.00E+00	5.38E-01	2.89E-03
GOTERM_BP_DIRECT	myoblast fusion	5	712	28	19512	1.79E-02	8.56E-01	4.89	1.00E+00	8.50E-01	3.12E-03
GOTERM_BP_DIRECT	positive regulation of synapse assembly	8	712	68	19512	1.17E-02	6.75E-01	3.22	1.00E+00	6.70E-01	3.24E-03
GOTERM_BP_DIRECT	response to xenobiotic stimulus	19	712	260	19512	7.13E-03	5.03E-01	2	1.00E+00	4.99E-01	3.31E-03
GOTERM_BP_DIRECT	negative chemotaxis	6	712	41	19512	1.61E-02	8.31E-01	4.01	1.00E+00	8.26E-01	3.50E-03

GOTERM_BP_DIRECT	low-density lipoprotein particle clearance	4	712	18	19512	2.61E-02	1.00E+00	6.09	1.00E+00	9.94E-01	3.57E-03
GOTERM_BP_DIRECT	multicellular organismal-level iron ion homeostasis	5	712	29	19512	2.01E-02	8.81E-01	4.72	1.00E+00	8.75E-01	3.66E-03
GOTERM_BP_DIRECT	regulation of synapse assembly	5	712	29	19512	2.01E-02	8.81E-01	4.72	1.00E+00	8.75E-01	3.66E-03
GOTERM_BP_DIRECT	spleen development	6	712	42	19512	1.77E-02	8.56E-01	3.91	1.00E+00	8.50E-01	3.96E-03
GOTERM_BP_DIRECT	regulation of cell shape	12	712	136	19512	1.11E-02	6.58E-01	2.42	1.00E+00	6.54E-01	4.17E-03
GOTERM_BP_DIRECT	cell differentiation	57	712	1094	19512	6.49E-03	4.77E-01	1.43	1.00E+00	4.74E-01	4.37E-03
GOTERM_BP_DIRECT	digestive tract morphogenesis	4	712	19	19512	3.02E-02	1.00E+00	5.77	1.00E+00	9.94E-01	4.40E-03
GOTERM_BP_DIRECT	fibrinolysis	4	712	19	19512	3.02E-02	1.00E+00	5.77	1.00E+00	9.94E-01	4.40E-03
GOTERM_BP_DIRECT	inner dynein arm assembly	4	712	19	19512	3.02E-02	1.00E+00	5.77	1.00E+00	9.94E-01	4.40E-03
GOTERM_BP_DIRECT	negative regulation of canonical Wnt signaling pathway	13	712	156	19512	1.19E-02	6.75E-01	2.28	1.00E+00	6.70E-01	4.77E-03
GOTERM_BP_DIRECT	central nervous system projection neuron axonogenesis	3	712	10	19512	4.91E-02	1.00E+00	8.22	1.00E+00	9.94E-01	4.79E-03
GOTERM_BP_DIRECT	axonogenesis involved in innervation	3	712	10	19512	4.91E-02	1.00E+00	8.22	1.00E+00	9.94E-01	4.79E-03
GOTERM_BP_DIRECT	cell-cell junction maintenance	3	712	10	19512	4.91E-02	1.00E+00	8.22	1.00E+00	9.94E-01	4.79E-03
GOTERM_BP_DIRECT	heparan sulfate proteoglycan catabolic process	3	712	10	19512	4.91E-02	1.00E+00	8.22	1.00E+00	9.94E-01	4.79E-03
GOTERM_BP_DIRECT	membranous septum morphogenesis	3	712	10	19512	4.91E-02	1.00E+00	8.22	1.00E+00	9.94E-01	4.79E-03
GOTERM_BP_DIRECT	regulation of ERK1 and ERK2 cascade	5	712	31	19512	2.52E-02	1.00E+00	4.42	1.00E+00	9.94E-01	4.93E-03
GOTERM_BP_DIRECT	cardiac muscle contraction	6	712	44	19512	2.13E-02	9.20E-01	3.74	1.00E+00	9.14E-01	5.01E-03
GOTERM_BP_DIRECT	monoatomic ion transport	38	712	672	19512	8.47E-03	5.42E-01	1.55	1.00E+00	5.38E-01	5.23E-03
GOTERM_BP_DIRECT	protein localization to synapse	4	712	20	19512	3.46E-02	1.00E+00	5.48	1.00E+00	9.94E-01	5.34E-03
GOTERM_BP_DIRECT	innervation	4	712	20	19512	3.46E-02	1.00E+00	5.48	1.00E+00	9.94E-01	5.34E-03
GOTERM_BP_DIRECT	positive regulation of epithelial cell proliferation	8	712	74	19512	1.81E-02	8.56E-01	2.96	1.00E+00	8.50E-01	5.46E-03
GOTERM_BP_DIRECT	phospholipid biosynthetic process	7	712	59	19512	2.01E-02	8.81E-01	3.25	1.00E+00	8.75E-01	5.49E-03
GOTERM_BP_DIRECT	endochondral ossification	5	712	32	19512	2.80E-02	1.00E+00	4.28	1.00E+00	9.94E-01	5.67E-03
GOTERM_BP_DIRECT	social behavior	7	712	60	19512	2.17E-02	9.25E-01	3.2	1.00E+00	9.19E-01	6.03E-03

GOTERM_BP_DIRECT	cell surface receptor protein tyrosine kinase signaling pathway	11	712	125	19512	1.61E-02	8.31E-01	2.41	1.00E+00	8.26E-01	6.08E-03
GOTERM_BP_DIRECT	cellular response to starvation	9	712	92	19512	1.90E-02	8.64E-01	2.68	1.00E+00	8.58E-01	6.39E-03
GOTERM_BP_DIRECT	renal absorption	3	712	11	19512	5.86E-02	1.00E+00	7.47	1.00E+00	9.94E-01	6.41E-03
GOTERM_BP_DIRECT	regulation of fatty acid metabolic process	3	712	11	19512	5.86E-02	1.00E+00	7.47	1.00E+00	9.94E-01	6.41E-03
GOTERM_BP_DIRECT	regulation of cell cycle phase transition	3	712	11	19512	5.86E-02	1.00E+00	7.47	1.00E+00	9.94E-01	6.41E-03
GOTERM_BP_DIRECT	negative regulation of inflammatory response to wounding	3	712	11	19512	5.86E-02	1.00E+00	7.47	1.00E+00	9.94E-01	6.41E-03
GOTERM_BP_DIRECT	iron ion transport	5	712	33	19512	3.10E-02	1.00E+00	4.15	1.00E+00	9.94E-01	6.49E-03
GOTERM_BP_DIRECT	non-canonical Wnt signaling pathway	5	712	33	19512	3.10E-02	1.00E+00	4.15	1.00E+00	9.94E-01	6.49E-03
GOTERM_BP_DIRECT	neuron projection development	13	712	162	19512	1.57E-02	8.31E-01	2.2	1.00E+00	8.26E-01	6.53E-03
GOTERM_BP_DIRECT	positive regulation of excitatory postsynaptic potential	5	712	34	19512	3.41E-02	1.00E+00	4.03	1.00E+00	9.94E-01	7.38E-03
GOTERM_BP_DIRECT	platelet-derived growth factor receptor signaling pathway	5	712	34	19512	3.41E-02	1.00E+00	4.03	1.00E+00	9.94E-01	7.38E-03
GOTERM_BP_DIRECT	pattern recognition receptor signaling pathway	4	712	22	19512	4.43E-02	1.00E+00	4.98	1.00E+00	9.94E-01	7.61E-03
GOTERM_BP_DIRECT	middle ear morphogenesis	4	712	22	19512	4.43E-02	1.00E+00	4.98	1.00E+00	9.94E-01	7.61E-03
GOTERM_BP_DIRECT	cilium movement	6	712	48	19512	2.98E-02	1.00E+00	3.43	1.00E+00	9.94E-01	7.70E-03
GOTERM_BP_DIRECT	negative regulation of cold-induced thermogenesis	6	712	48	19512	2.98E-02	1.00E+00	3.43	1.00E+00	9.94E-01	7.70E-03
GOTERM_BP_DIRECT	outflow tract morphogenesis	7	712	63	19512	2.69E-02	1.00E+00	3.04	1.00E+00	9.94E-01	7.87E-03
GOTERM_BP_DIRECT	negative regulation of fibrinolysis	3	712	12	19512	6.87E-02	1.00E+00	6.85	1.00E+00	9.94E-01	8.32E-03
GOTERM_BP_DIRECT	cochlea development	5	712	35	19512	3.74E-02	1.00E+00	3.91	1.00E+00	9.94E-01	8.36E-03
GOTERM_BP_DIRECT	mRNA transcription by RNA polymerase II	6	712	49	19512	3.23E-02	1.00E+00	3.36	1.00E+00	9.94E-01	8.51E-03
GOTERM_BP_DIRECT	extrinsic apoptotic signaling pathway in absence of ligand	5	712	36	19512	4.10E-02	1.00E+00	3.81	1.00E+00	9.94E-01	9.42E-03
GOTERM_BP_DIRECT	cytokine-mediated signaling pathway	12	712	153	19512	2.45E-02	1.00E+00	2.15	1.00E+00	9.94E-01	1.04E-02
GOTERM_BP_DIRECT	retinoic acid receptor signaling pathway	4	712	24	19512	5.52E-02	1.00E+00	4.57	1.00E+00	9.94E-01	1.04E-02

GOTERM_BP_DIRECT	biosynthetic process	3	712	13	19512	7.93E-02	1.00E+00	6.32	1.00E+00	9.94E-01	1.05E-02
GOTERM_BP_DIRECT	synaptic signaling	3	712	13	19512	7.93E-02	1.00E+00	6.32	1.00E+00	9.94E-01	1.05E-02
GOTERM_BP_DIRECT	positive regulation of platelet activation	3	712	13	19512	7.93E-02	1.00E+00	6.32	1.00E+00	9.94E-01	1.05E-02
GOTERM_BP_DIRECT	cellular response to leucine starvation	3	712	13	19512	7.93E-02	1.00E+00	6.32	1.00E+00	9.94E-01	1.05E-02
GOTERM_BP_DIRECT	long-term memory	5	712	37	19512	4.46E-02	1.00E+00	3.7	1.00E+00	9.94E-01	1.06E-02
GOTERM_BP_DIRECT	endodermal cell differentiation	5	712	37	19512	4.46E-02	1.00E+00	3.7	1.00E+00	9.94E-01	1.06E-02
GOTERM_BP_DIRECT	post-embryonic development	8	712	84	19512	3.35E-02	1.00E+00	2.61	1.00E+00	9.94E-01	1.15E-02
GOTERM_BP_DIRECT	positive regulation of cytokine production involved in inflammatory response	5	712	38	19512	4.85E-02	1.00E+00	3.61	1.00E+00	9.94E-01	1.18E-02
GOTERM_BP_DIRECT	intermembrane lipid transfer	5	712	38	19512	4.85E-02	1.00E+00	3.61	1.00E+00	9.94E-01	1.18E-02
GOTERM_BP_DIRECT	negative regulation of inflammatory response	12	712	156	19512	2.78E-02	1.00E+00	2.11	1.00E+00	9.94E-01	1.20E-02
GOTERM_BP_DIRECT	phosphatidylcholine biosynthetic process	4	712	25	19512	6.11E-02	1.00E+00	4.38	1.00E+00	9.94E-01	1.21E-02
GOTERM_BP_DIRECT	response to electrical stimulus	4	712	25	19512	6.11E-02	1.00E+00	4.38	1.00E+00	9.94E-01	1.21E-02
GOTERM_BP_DIRECT	positive regulation of canonical Wnt signaling pathway	10	712	120	19512	3.14E-02	1.00E+00	2.28	1.00E+00	9.94E-01	1.25E-02
GOTERM_BP_DIRECT	skeletal muscle tissue development	7	712	69	19512	3.96E-02	1.00E+00	2.78	1.00E+00	9.94E-01	1.27E-02
GOTERM_BP_DIRECT	cellular response to UV-B	3	712	14	19512	9.04E-02	1.00E+00	5.87	1.00E+00	9.94E-01	1.30E-02
GOTERM_BP_DIRECT	enamel mineralization	3	712	14	19512	9.04E-02	1.00E+00	5.87	1.00E+00	9.94E-01	1.30E-02
GOTERM_BP_DIRECT	sphingolipid catabolic process	3	712	14	19512	9.04E-02	1.00E+00	5.87	1.00E+00	9.94E-01	1.30E-02
GOTERM_BP_DIRECT	cytoskeleton-dependent intracellular transport	3	712	14	19512	9.04E-02	1.00E+00	5.87	1.00E+00	9.94E-01	1.30E-02
GOTERM_BP_DIRECT	phosphatidylcholine catabolic process	3	712	14	19512	9.04E-02	1.00E+00	5.87	1.00E+00	9.94E-01	1.30E-02
GOTERM_BP_DIRECT	nerve growth factor signaling pathway	3	712	14	19512	9.04E-02	1.00E+00	5.87	1.00E+00	9.94E-01	1.30E-02
GOTERM_BP_DIRECT	cell morphogenesis	10	712	121	19512	3.29E-02	1.00E+00	2.26	1.00E+00	9.94E-01	1.32E-02
GOTERM_BP_DIRECT	negative regulation of ERK1 and ERK2 cascade	8	712	86	19512	3.73E-02	1.00E+00	2.55	1.00E+00	9.94E-01	1.32E-02

GOTERM_BP_DIRECT	homeostasis of number of cells within a tissue	5	712	39	19512	5.26E-02	1.00E+00	3.51	1.00E+00	9.94E-01	1.32E-02
GOTERM_BP_DIRECT	intracellular receptor signaling pathway	5	712	39	19512	5.26E-02	1.00E+00	3.51	1.00E+00	9.94E-01	1.32E-02
GOTERM_BP_DIRECT	tissue development	6	712	54	19512	4.63E-02	1.00E+00	3.04	1.00E+00	9.94E-01	1.35E-02
GOTERM_BP_DIRECT	phospholipid metabolic process	7	712	70	19512	4.20E-02	1.00E+00	2.74	1.00E+00	9.94E-01	1.37E-02
GOTERM_BP_DIRECT	ventricular cardiac muscle tissue morphogenesis	4	712	26	19512	6.73E-02	1.00E+00	4.22	1.00E+00	9.94E-01	1.39E-02
GOTERM_BP_DIRECT	response to estradiol	8	712	87	19512	3.94E-02	1.00E+00	2.52	1.00E+00	9.94E-01	1.41E-02
GOTERM_BP_DIRECT	regulation of blood pressure	8	712	87	19512	3.94E-02	1.00E+00	2.52	1.00E+00	9.94E-01	1.41E-02
GOTERM_BP_DIRECT	regulation of endocytosis	5	712	40	19512	5.68E-02	1.00E+00	3.43	1.00E+00	9.94E-01	1.46E-02
GOTERM_BP_DIRECT	visual learning	6	712	55	19512	4.94E-02	1.00E+00	2.99	1.00E+00	9.94E-01	1.47E-02
GOTERM_BP_DIRECT	roof of mouth development	7	712	71	19512	4.45E-02	1.00E+00	2.7	1.00E+00	9.94E-01	1.48E-02
GOTERM_BP_DIRECT	response to hypoxia	13	712	181	19512	3.36E-02	1.00E+00	1.97	1.00E+00	9.94E-01	1.56E-02
GOTERM_BP_DIRECT	epithelial cell proliferation	7	712	72	19512	4.72E-02	1.00E+00	2.66	1.00E+00	9.94E-01	1.58E-02
GOTERM_BP_DIRECT	positive regulation of mesenchymal cell proliferation	4	712	27	19512	7.38E-02	1.00E+00	4.06	1.00E+00	9.94E-01	1.58E-02
GOTERM_BP_DIRECT	phospholipid translocation	4	712	27	19512	7.38E-02	1.00E+00	4.06	1.00E+00	9.94E-01	1.58E-02
GOTERM_BP_DIRECT	positive regulation of oligodendrocyte differentiation	4	712	27	19512	7.38E-02	1.00E+00	4.06	1.00E+00	9.94E-01	1.58E-02
GOTERM_BP_DIRECT	regulation of cardiac muscle contraction by regulation of the release of sequestered calcium ion	4	712	27	19512	7.38E-02	1.00E+00	4.06	1.00E+00	9.94E-01	1.58E-02
GOTERM_BP_DIRECT	odontogenesis of dentin-containing tooth	6	712	56	19512	5.27E-02	1.00E+00	2.94	1.00E+00	9.94E-01	1.60E-02
GOTERM_BP_DIRECT	regulation of GTPase activity	5	712	41	19512	6.12E-02	1.00E+00	3.34	1.00E+00	9.94E-01	1.61E-02
GOTERM_BP_DIRECT	collagen catabolic process	5	712	41	19512	6.12E-02	1.00E+00	3.34	1.00E+00	9.94E-01	1.61E-02
GOTERM_BP_DIRECT	response to nicotine	5	712	41	19512	6.12E-02	1.00E+00	3.34	1.00E+00	9.94E-01	1.61E-02
GOTERM_BP_DIRECT	positive regulation of inflammatory response	10	712	125	19512	3.93E-02	1.00E+00	2.19	1.00E+00	9.94E-01	1.63E-02
GOTERM_BP_DIRECT	neuron projection morphogenesis	7	712	73	19512	4.99E-02	1.00E+00	2.63	1.00E+00	9.94E-01	1.70E-02
GOTERM_BP_DIRECT	cellular response to retinoic acid	7	712	73	19512	4.99E-02	1.00E+00	2.63	1.00E+00	9.94E-01	1.70E-02
GOTERM_BP_DIRECT	response to lipopolysaccharide	12	712	164	19512	3.80E-02	1.00E+00	2.01	1.00E+00	9.94E-01	1.73E-02

GOTERM_BP_DIRECT	lung development	9	712	109	19512	4.57E-02	1.00E+00	2.26	1.00E+00	9.94E-01	1.83E-02
GOTERM_BP_DIRECT	neurotransmitter transport	6	712	58	19512	5.97E-02	1.00E+00	2.83	1.00E+00	9.94E-01	1.87E-02
GOTERM_BP_DIRECT	lipid homeostasis	6	712	58	19512	5.97E-02	1.00E+00	2.83	1.00E+00	9.94E-01	1.87E-02
GOTERM_BP_DIRECT	embryonic hindlimb morphogenesis	4	712	29	19512	8.74E-02	1.00E+00	3.78	1.00E+00	9.94E-01	2.02E-02
GOTERM_BP_DIRECT	cholesterol transport	4	712	29	19512	8.74E-02	1.00E+00	3.78	1.00E+00	9.94E-01	2.02E-02
GOTERM_BP_DIRECT	cochlea morphogenesis	4	712	29	19512	8.74E-02	1.00E+00	3.78	1.00E+00	9.94E-01	2.02E-02
GOTERM_BP_DIRECT	epithelial to mesenchymal transition	6	712	60	19512	6.71E-02	1.00E+00	2.74	1.00E+00	9.94E-01	2.18E-02
GOTERM_BP_DIRECT	positive regulation of epithelial to mesenchymal transition	6	712	60	19512	6.71E-02	1.00E+00	2.74	1.00E+00	9.94E-01	2.18E-02
GOTERM_BP_DIRECT	positive regulation of phagocytosis	6	712	60	19512	6.71E-02	1.00E+00	2.74	1.00E+00	9.94E-01	2.18E-02
GOTERM_BP_DIRECT	gamma-aminobutyric acid signaling pathway	4	712	30	19512	9.46E-02	1.00E+00	3.65	1.00E+00	9.94E-01	2.27E-02
GOTERM_BP_DIRECT	toll-like receptor signaling pathway	4	712	30	19512	9.46E-02	1.00E+00	3.65	1.00E+00	9.94E-01	2.27E-02
GOTERM_BP_DIRECT	establishment or maintenance of epithelial cell apical/basal polarity	4	712	30	19512	9.46E-02	1.00E+00	3.65	1.00E+00	9.94E-01	2.27E-02
GOTERM_BP_DIRECT	positive regulation of transcription by RNA polymerase II	59	712	1243	19512	3.14E-02	1.00E+00	1.3	1.00E+00	9.94E-01	2.31E-02
GOTERM_BP_DIRECT	sensory perception of sound	12	712	171	19512	4.88E-02	1.00E+00	1.92	1.00E+00	9.94E-01	2.32E-02
GOTERM_BP_DIRECT	pattern specification process	6	712	61	19512	7.10E-02	1.00E+00	2.7	1.00E+00	9.94E-01	2.35E-02
GOTERM_BP_DIRECT	epithelial cell differentiation	9	712	114	19512	5.68E-02	1.00E+00	2.16	1.00E+00	9.94E-01	2.37E-02
GOTERM_BP_DIRECT	negative regulation of cell migration	13	712	193	19512	5.06E-02	1.00E+00	1.85	1.00E+00	9.94E-01	2.51E-02
GOTERM_BP_DIRECT	defense response	12	712	173	19512	5.23E-02	1.00E+00	1.9	1.00E+00	9.94E-01	2.51E-02
GOTERM_BP_DIRECT	negative regulation of phosphatidylinositol 3-kinase/protein kinase B signal transduction	7	712	79	19512	6.81E-02	1.00E+00	2.43	1.00E+00	9.94E-01	2.51E-02
GOTERM_BP_DIRECT	negative regulation of epithelial cell proliferation	6	712	62	19512	7.51E-02	1.00E+00	2.65	1.00E+00	9.94E-01	2.52E-02
GOTERM_BP_DIRECT	fatty acid metabolic process	14	712	214	19512	5.03E-02	1.00E+00	1.79	1.00E+00	9.94E-01	2.56E-02
GOTERM_BP_DIRECT	positive regulation of cell population proliferation	29	712	542	19512	3.98E-02	1.00E+00	1.47	1.00E+00	9.94E-01	2.59E-02
GOTERM_BP_DIRECT	sodium ion transmembrane transport	10	712	135	19512	5.88E-02	1.00E+00	2.03	1.00E+00	9.94E-01	2.62E-02
GOTERM_BP_DIRECT	animal organ morphogenesis	10	712	136	19512	6.10E-02	1.00E+00	2.02	1.00E+00	9.94E-01	2.74E-02

GOTERM_BP_DIRECT	regulation of postsynapse assembly	5	712	47	19512	9.12E-02	1.00E+00	2.92	1.00E+00	9.94E-01	2.77E-02
GOTERM_BP_DIRECT	response to nutrient levels	7	712	81	19512	7.50E-02	1.00E+00	2.37	1.00E+00	9.94E-01	2.83E-02
GOTERM_BP_DIRECT	intracellular signal transduction	27	712	501	19512	4.48E-02	1.00E+00	1.48	1.00E+00	9.94E-01	2.88E-02
GOTERM_BP_DIRECT	regulation of cell adhesion	6	712	64	19512	8.35E-02	1.00E+00	2.57	1.00E+00	9.94E-01	2.89E-02
GOTERM_BP_DIRECT	actin cytoskeleton organization	15	712	239	19512	5.55E-02	1.00E+00	1.72	1.00E+00	9.94E-01	2.96E-02
GOTERM_BP_DIRECT	embryo implantation	5	712	48	19512	9.68E-02	1.00E+00	2.85	1.00E+00	9.94E-01	3.00E-02
GOTERM_BP_DIRECT	gene expression	17	712	282	19512	5.46E-02	1.00E+00	1.65	1.00E+00	9.94E-01	3.03E-02
GOTERM_BP_DIRECT	collagen fibril organization	6	712	65	19512	8.79E-02	1.00E+00	2.53	1.00E+00	9.94E-01	3.09E-02
GOTERM_BP_DIRECT	heart morphogenesis	6	712	66	19512	9.24E-02	1.00E+00	2.49	1.00E+00	9.94E-01	3.30E-02
GOTERM_BP_DIRECT	modulation of chemical synaptic transmission	9	712	123	19512	8.06E-02	1.00E+00	2.01	1.00E+00	9.94E-01	3.63E-02
GOTERM_BP_DIRECT	positive regulation of JNK cascade	8	712	104	19512	8.56E-02	1.00E+00	2.11	1.00E+00	9.94E-01	3.65E-02
GOTERM_BP_DIRECT	endocytosis	14	712	226	19512	7.11E-02	1.00E+00	1.7	1.00E+00	9.94E-01	3.81E-02
GOTERM_BP_DIRECT	regulation of actin cytoskeleton organization	8	712	105	19512	8.89E-02	1.00E+00	2.09	1.00E+00	9.94E-01	3.83E-02
GOTERM_BP_DIRECT	positive regulation of gene expression	27	712	515	19512	5.80E-02	1.00E+00	1.44	1.00E+00	9.94E-01	3.85E-02
GOTERM_BP_DIRECT	canonical Wnt signaling pathway	8	712	106	19512	9.24E-02	1.00E+00	2.07	1.00E+00	9.94E-01	4.01E-02
GOTERM_BP_DIRECT	axonogenesis	9	712	126	19512	8.97E-02	1.00E+00	1.96	1.00E+00	9.94E-01	4.13E-02
GOTERM_BP_DIRECT	sodium ion transport	10	712	146	19512	8.63E-02	1.00E+00	1.88	1.00E+00	9.94E-01	4.16E-02
GOTERM_BP_DIRECT	anatomical structure morphogenesis	11	712	170	19512	9.31E-02	1.00E+00	1.77	1.00E+00	9.94E-01	4.75E-02
