# Supplementary material for: Establishment of a Common Marmoset Lineage Carrying a Frameshift Mutation in SETD1A, a Schizophrenia Risk Gene
Source: J Neurochem. 2026 Sep 3;170(9):e70546. doi: 10.1111/jnc.70546 (PMC13539942; doi:10.1111/jnc.70546)
Supplement: Supplementary file 1 [file JNC-170-e70546-s001.zip › JNC_70546__pe.docx]

**Figure S1** The whole membrane images of the Western blot analyses performed in this study are

shown (WT: wild-type, #630: the *SETD1A* F1 mutant. n = 3 replicate lanes from whole protein

sample extracted from the same culture dishes). Chemiluminescence images and colorimetric

images were overlayed. (A) Immunoblotting for SETD1A (upper) and LMNB1 (lower). The HiMarkTM

Pre-stained Protein Standard (Thermo Fisher Scientific, cat. no. LC5699) was used as a molecular

weight marker. (B) Immunoblotting for H3K4me3 (upper) and Histone H3 (lower). The BenchMarkTM

Protein Ladder (Thermo Fisher Scientific, cat. no. 10747012) was used as a molecular weight

marker.

**Figure S2** Principal component analysis (PCA) of the CUT&Tag (left) and RNA-seq (right) datasets

generated in this study. The proportion of variance explained by each principal component is shown

in the axis labels. Each data point represents a technical replicate (black: wild-type, white: the

*SETD1A* F1 mutant (#630). n = 3 technical replicates).

Table S1 Information of the *SETD1A* F1 mutant #630 and all used wild-type marmosets.

Table S2 Details of embryo manipulation for each *SETD1A* F0 mutant.

Table S3 GO terms associated with downregulated DEGs without H3K4me3-loss.

Table S4 GO terms associated with upregulated DEGs without H3K4me3-gain.
